# Statistics is not measurement: The inbuilt semantics of psychometric scales and language-based models obscures crucial epistemic differences

**DOI:** 10.3389/fpsyg.2025.1534270

**Published:** 2025-06-26

**Authors:** Jana Uher

**Affiliations:** School of Human Sciences, University of Greenwich, London, United Kingdom

**Keywords:** measurement, psychometrics, large language models (LLMs), natural language processing (NLP), rating scales, modelling relation, semantics-syntax, metrology

## Abstract

This article provides a comprehensive critique of psychology's overreliance on statistical modelling at the expense of epistemologically grounded measurement processes. It highlights that statistics deals with structural relations in data regardless of what these data represent, whereas measurement establishes traceable empirical relations between the phenomena studied and the data representing information about them. These crucial epistemic differences are elaborated using Rosen's general model of measurement, involving the coherent modelling of the (1) objects of research, (2) data generation (encoding), (3) formal manipulation (e.g., statistical analysis) and (4) result interpretation regarding the objects studied (decoding). This system of interrelated modelling relations is shown to underlie metrologists' approaches for tackling the problem of epistemic circularity in physical measurement, illustrated in the special cases of measurement coordination and calibration. The article then explicates psychology's challenges for establishing genuine analogues of measurement, which arise from the peculiarities of its study phenomena (e.g., higher-order complexity, non-ergodicity) and language-based methods (e.g., inbuilt semantics). It demonstrates that psychometrics cannot establish coordinated and calibrated modelling relations, thus generating only pragmatic quantifications with predictive power but precluding epistemically justified inferences on the phenomena studied. This epistemic gap is often overlooked, however, because many psychologists mistake their methods' inbuilt semantics—thus, descriptions of their study phenomena (e.g., in rating scales, item variables, statistical models)—for the phenomena described. This blurs the epistemically necessary distinction between the phenomena studied and those used as means of investigation, thereby confusing ontological with epistemological concepts—psychologists' cardinal error. Therefore, many mistake judgements of verbal statements for measurements of the phenomena described and overlook that statistics can neither establish nor analyze a model's relations to the phenomena explored. The article elaborates epistemological and methodological fundamentals to establish coherent modelling relations between real and formal study system and to distinguish the epistemic components involved, considering psychology's peculiarities. It shows that epistemically justified inferences necessitate methods for analysing individuals' unrestricted verbal responses, now advanced through artificial intelligence systems modelling natural language (e.g., NLP algorithms, LLMs). Their increasing use to generate standardised descriptions of study phenomena for rating scales and constructs, by contrast, will only perpetuate psychologists' cardinal error—and thus, psychology's crisis.

## 1 Statistics vs. measurement

Psychology cherishes its sophisticated ‘measurement' and modelling techniques for enabling quantitative research—the hallmark of modern science. A closer look reveals, however, that only methods of statistical data analysis are well elaborated, which together with pertinent research designs (e.g., between-subjects) fill our books and journals on psychological research methods. This emphasis reflects the prevailing view that statistics constitutes psychology's approach for ‘measuring' its non-observable study phenomena (e.g., in psychometrics). This assumption, however, is based on epistemic errors because statistics neither *is* measurement nor is statistics necessary for measurement.

### 1.1 Different scientific activities for different epistemic purposes

Measurement and measurement scales have been successfully developed in physics and metrology—the science of physical measurement and its application (JCGM100:2008, 2008, p. 2.2)—long before statistics was invented (Abran et al., 2012; Fisher, 2009; Uher, 2022b, 2023a). Measurement and statistics involve different scientific activities designed for different epistemic (knowledge-related) purposes.

*Measurement* requires traceable empirical interactions with the specific quantities to be measured in the phenomena and properties under study—the *measurands* (e.g., person A's body temperature but not A's body weight or volume; person B's duration of speaking in a specific situation). Epistemically justifiable inferences from observable indications of these empirical interactions back to the measurands require theoretical knowledge about both the object of research and the objects used as measuring instruments as well as their conceptualisation in a defined process structure within a realist framework (Mari et al., 2021; Schrödinger, 1964; von Neumann, 1955). Its empirical implementation necessitates unbroken documented connection chains that establish proportional (quantitative) relations of the results with both (1) the measurand's unknown quantity (e.g., A's body temperature; B's duration of speaking)—the principle of *data generation traceability*—and (2) a known reference quantity (e.g., international units). This reference is necessary to establish the results' quantitative meaning regarding the specific property studied (e.g., *how* warm or *how* long that is)—the principle of *numerical traceability* (Uher, 2018a, 2020b, 2021c,d, 2022a,b, 2023a). Process structures thus-established allow for deriving epistemically justified information about specific quantities that are assumed to exist in an object of research and for representing this information in sign systems that are unambiguously interpretable regarding those measurands (e.g., ‘*T*_*Pers*_*A*_ = 36.9°C'; ‘*d*_*Pers*_*B*_ = 16.2 mins/h').

*Statistics*, by contrast, enables probabilistic descriptions of what might happen as a consequence of complex, poorly understood and possibly random events and processes as well as of constraints that are set by stochastic boundaries (e.g., distribution curves). In data sets, statistical methods allow us to identify regularities beyond pure randomness, to group cases and compare groups by their parameters, to model and extrapolate patterns as well as to estimate error and uncertainty for justifying inferences from samples to distribution patterns in hypothetical populations (Romeijn, 2017). Statistics builds on theories that define the workings of the analytical operations performed (e.g., mathematical statistics, probability theory, item response theory). But it does not build on theories about the objects of research that scientists may aim to analyse for prevalences, differences and trends, and that may be as diverse as diseases, therapeutic treatments, behaviours, intellectual abilities, financial markets, policies and others. Statistics is mute about the specific phenomena and properties analysed (Strauch, 1976). That is, statistics concerns the analysis of data sets *regardless of what these data are meant to represent*. Therefore, it does not require a term denoting the specific quantity to be measured in the real study objects—the measurand. This may explain why most psychologists are unfamiliar with this basic term. Their focus on ‘true scores' in statistical modelling obscures the epistemic distinction between the real quantity to be measured and the measurement results used to estimate it (Strom and Tabatadze, 2022).

Statistics, however, is fundamental to so-called psychological ‘measurement'. Why?

### 1.2 Psychological ‘measurement': Statistical analysis enabling pragmatic quantification

Psychological ‘measurement' (e.g., psychometrics) is aimed at discriminating well and consistently between cases (e.g., individuals, groups) and in ways considered important (e.g., social relevance, relations to future outcomes). Therefore, ‘measuring instruments' (e.g., intelligence tests, rating ‘scales') are designed such as to generate data structures that are useful for these purposes (e.g., specific distribution or association patterns). To this pragmatic end, statistical analyses are indispensable (Uher, 2021c).

Many psychologists believe that measurement involves the assignment of numbers and capitalises on their mathematically defined quantitative meaning. In measurement, however, we assign numerical values whose specific *quantitative meaning* is conventionally agreed and traceable to defined reference quantities (e.g., of the International System, SI; BIPM, 2019). We know this from everyday life. The numerical values of ‘1 kilogram', ‘2.205 pounds', ‘35.274 ounces' and ‘0.1575 stones' differ—but they all indicate the same quantity of weight. These differences originate from once arbitrary decisions on specific quantities that were used as references. Meanwhile, their specific quantitative meaning is conventionally agreed and indicated by the measurement unit (e.g., ‘kg', ‘lb', ‘oz', ‘st'). The unit also indicates the specific kind of property measured—‘1' ‘kilogram' is not ‘1' ‘litre', ‘1' ‘metre' or ‘1' ‘volt'. That is, the measurement unit specifies also a result's *qualitative meaning*, such as whether it is a quantity of weight, volume, length or electric potential.

In psychology, by contrast, ‘measurement' values are commonly presented without a unit, thus indicating neither specific qualities (e.g., frequency, intensity or level of agreement) nor specific quantities of them (e.g., *how* often or *how* much of that). Unit-free values—therefore called ‘scores'—are meaningless in themselves. It requires statistics to first create quantitative meaning for scores from their distribution patterns and interrelations within specific samples (e.g., differential comparisons within age groups), leading to reference group effects (Uher, 2021c,d, 2022a, 2023a). Hence, psychometric scores constitute *quantifications that are created for specific uses, contexts and pragmatic purposes*, such as for making decisions or projections in applied settings (Barrett, 2003; Dawes et al., 1989; Newfield et al., 2022). This highlights first important differences from genuine measurement.

Specifically, psychometric theories and empirical practices clearly build on a *pragmatic utilitarian framework* that is aimed at producing quantitative results with statistically desirable and practically useful structures. By contrast, traceable relations to empirical interactions with the quantities to be measured (measurands) in individuals and to known reference quantities are neither conceptualised nor empirically implemented. Nevertheless, psychometricians explicitly aim for “measuring the mind” (Borsboom, 2005)—thus, for ‘measuring' specific quantitative properties that individuals are assumed to possess. Accordingly, psychometric results (e.g., IQ scores) are interpreted as quantifications of the studied individuals' psychical^1^ properties (e.g., intellectual abilities) and used for making decisions about these individuals (e.g., education). Here, psychometricians clearly invoke the *realist framework* underlying physical measurement, ignoring that they have theoretically and empirically established instead only a pragmatic utilitarian framework (Uher, 2021c,d, 2022b, 2023a). This confusion of two incompatible epistemological frameworks entails numerous conceptual and logical errors, as this article will show (Section 3).

But regardless of this, psychometricians' declared aims and result interpretations highlight basic ideas of measurement that are shared by metrologists, physicists and psychologists alike. These ideas can be formulated as two *epistemic criteria* as the most basic common denominators considered across the sciences that characterise an empirical process as one of measurement. Criterion 1 is the *epistemically justified attribution* of the generated quantitative results to the specific properties to be measured (measurands) in the study phenomena and to nothing else. Criterion 2 is the *public interpretability* of the results' quantitative meaning with regard to those measurands (Uher, 2020b, 2021a,b, 2023a). These two criteria are key to distinguish genuine measurement from other processes of quantification (e.g., opinions, judgements, evaluations). Importantly, this is not to classify some approaches as ‘superior' or ‘inferior'. Rather, a criterion-based approach to define measurement is essential for scrutinising the epistemic fundamentals of a field's pertinent theories and practices. This allowed for identifying, for example, the epistemological inconsistencies inherent to psychometrics (Uher, 2021c,d). A criterion-based approach is also crucial for pinpointing commonalities and differences between sciences.

Concretely, it shows that proposals to ‘soften', ‘weaken' or ‘widen' the definition of measurement for psychology (Eronen, 2024; Finkelstein, 2003; Mari et al., 2015) are epistemically mistaken. Certainly, psychology does not need the high levels of measurement accuracy and precision, as necessary for sciences like physics, chemistry and medicine where errors can lead to the collapse of buildings, chemical explosions or drug overdoses. But changing the definition of a scientific activity as fundamental to empirical science as that of measurement cannot establish its comparability across sciences. Much in contrast, it undermines comparability because it fails to provide guiding principles that specify how analogues of measurement that appropriately consider the study phenomena's peculiarities can be implemented in other sciences. The methodological principles of data generation traceability and numerical traceability, for example, can guide the design of discipline-specific processes that allow for meeting the two epistemic criteria of measurement also in psychology (Uher, 2018a, 2020b, 2022a,b, 2023a). Labelling disparate procedures uniformly as ‘measurement' also obscures essential and necessary differences in the theories and practices established in different sciences as well as inevitable limitations. Ultimately, measurement is not just any activity to generate numerical data but involves defined processes that justify the high public trust placed in it (Abran et al., 2012; Porter, 1995).

In everyday life, the *differences between measurement and pragmatic quantification* are obvious. When we buy apples in a shop, we measure their weight. But we do not measure their price. The apples' weight is a quantitative property, which they possess as real physical objects. It is determined through their traceable empirical interaction with a measuring instrument (therefore, we must place the apples on the weighing scale). The specific quantity of weight that we denote as ‘1 kg' is (nowadays) specified through known reference quantities, which are internationally agreed and thus, universally interpretable. The apples' price, by contrast, is pragmatically quantified for various purposes within a given socio-economic system that go beyond the apples' specific physical properties (e.g., sales, profit). Thus, the price merely indicates an attributed quantitative value—an *attribute*—which therefore changes across contexts and times (e.g., supply, demand and tariffs). The price's specific quantitative meaning, in turn, is derived from its relations to other attributed socio-economic values (e.g., currency, inflation) and can therefore vary in itself as well.

Psychological ‘measurement' (e.g., psychometrics) is widely practised and justified for its pragmatic and utilitarian purposes. However, it does not involve genuine measurement as often claimed (therefore here put in inverted commas, as are the psychological terms ‘scales' and ‘instruments'^2^). Instead, psychological ‘measurement' serves other epistemic purposes for which statistics is indispensable. Its focus is on analysing structures in data sets, such as data on persons' test performances or responses to rating ‘scales', in order to derive hypothetical quantitative relations, such as levels of “person ability” or item difficulty in Rasch modelling and item response theory. But the specific ways in which the analysed data—as well as the performances and responses encoded in these data—are generated in the first place are still hardly studied (Lundmann and Villadsen, 2016; Rosenbaum and Valsiner, 2011; Toomela, 2008; Uher, 2015c, 2018a,b, 2021a, 2022a, 2023a; Uher and Visalberghi, 2016; Uher et al., 2013b; Wagoner and Valsiner, 2005).

Indeed, rating ‘scales', psychology's most widely-used method of *quantitative data generation*, remained largely unchanged since their invention a century ago (Likert, 1932; Thurstone, 1928). This is astounding given that rating data form the basis of much of the empirical evidence used to test scientific hypotheses and theories, to make decisions about individuals in applied settings (Uher, 2018a, 2022b, 2023a) and to evaluate the effectiveness of interventions and trainings (Truijens et al., 2019b).

Hence, there is a *gap* between psychologists' numerical data and statistically modelled quantitative results, on the one side, and the specific entities to be quantified in their actual study phenomena, on the other. Bridging this gap requires measurement.

### 1.3 Metrological frameworks of measurement: Inherent limitations for psychology

Unlike statistics, measurement concerns how the data are generated—thus, the ways in which they are empirically connected both with the unknown quantity to be measured (measurand) in the study phenomena (data generation traceability) and with known reference quantities (numerical traceability). Unbroken documented connection chains determine how the measurement results can be interpreted regarding these measurands qualitatively and quantitatively (epistemic criteria 1 and 2). These two traceability principles underlie the measurement processes established in metrology (Uher, 2020b, 2022a).

Metrology, however, is concerned solely with the measurement of physical properties in non-living nature that feature *invariant* relations. Such properties are *always related to one another in the same ways* (under specified conditions), such as the fundamental relations between electric voltage (V), current (I) and resistance (R). It is this peculiarity that enables their formalisation in *immutable laws* (e.g., Ohm's law) and non-contradictory *mathematical equations* (formulas, e.g., *V* = *I* ^*^ *R*). Invariant relations can also be codified in *natural constants* (e.g., gravity on Earth, speed of light) and internationally agreed systems of units (e.g., metric, imperial; JCGM100:2008, 2008). Therefore, physical laws and formulas, natural constants and international units of measurement are assumed to be *universally* applicable.

But precisely because of this peculiarity, metrological frameworks cannot be applied or translated to psychological research as directly as metrologists and psychometricians increasingly propose (e.g., Fisher and Pendrill, 2024; Mari et al., 2021). This is because psychology's objects of research feature peculiarities not known from the non-living ‘world'. These involve variability, change and novel properties emerging from *complex relations* leading to irreversible development as well as the non-physicality and abstract nature of experience, and others (Hartmann, 1964; Morin, 1992). Moreover, unlike physical sciences and metrology, psychology explores not just objects and relations of specific phenomena (e.g., behaviours) in themselves but also, and in particular, their *individual (subjective) and socio-cultural (inter-subjective)* perception, interpretation, apprehension and appraisal (Wundt, 1896). These complex study phenomena are described in *multi-referential conceptual systems—constructs*. These conceptual systems cannot be studied with physical measuring instruments but require language-based methods instead (Kelly, 1955; Uher, 2022b, 2023b). Language, however, involves complexities that present unparalleled challenges to standardised quantitative inquiry, as this article will demonstrate. To tackle the challenges posed by psychology's complex study phenomena and methods of inquiry, metrology provides neither conceptual nor methodological fundamentals (Uher, 2018a, 2020b, 2022a).

Attempts to directly apply a science's concepts and theories to study phenomena not explored by that science involve challenges that cannot be mastered using the conceptual and methodological fundamentals of just single disciplines. Such *interdisciplinary*^3^ approaches underlie the current attempts to directly apply or translate metrological concepts to psychological ‘measurement' and psychometrics (e.g., Fisher and Pendrill, 2024; Mari et al., 2021). But they overlook fundamental ontological, epistemological and methodological differences. Developing epistemically justified research frameworks that are applicable across the sciences in that they are appropriate to the peculiarities of their different objects of research requires scrutinising the basic presuppositions of all the sciences involved. Such elaborations are at the core of transdisciplinarity, which is therefore applied in this article.

### 1.4 Transdisciplinarity: A new way of thinking and scientific inquiry

*Transdisciplinarity* has gained recognition as a new way of thinking about and engaging in scientific inquiry (Montuori, 2008; Nicolescu, 2002, 2008). Unlike all other types of disciplinary collaboration (e.g., cross-, multi- and inter-^4^), transdisciplinarity is aimed at analysing complex systems and complex (“wicked”) real-world problems, at developing an understanding of the ‘world' in its complexity and at generating unitary intellectual frameworks beyond specific disciplinary perspectives. To enable such explorations, transdisciplinarity^5^ not only relies on disciplinary paradigms but also transcends and integrates them. It is aimed at exposing disciplinary boundaries to facilitate the understanding of implicit assumptions, processes of inquiry and resulting knowledge as well as to discover hidden connections between different disciplines and their respective bodies of knowledge. A key focus is on identifying non-obvious differences, particularly in the underlying *ontology* (philosophy and theory of being), *epistemology* (philosophy and theory of knowing) and *methodology* (philosophy and theory of methods, connecting abstract philosophy of science with empirical research). That is, transdisciplinarity explores research questions that can be comprehended only outside of the boundaries of separate disciplines and therefore challenges the entire framework of disciplinary thinking and knowledge organisation (Bernstein, 2015; Gibbs and Beavis, 2020; Piaget, 1972; Pohl, 2011; Uher, 2024).

The present analyses—spanning concepts and approaches from psychology, social sciences, life sciences, physical sciences and metrology—rely on the *Transdisciplinary Philosophy-of-Science Paradigm for Research on Individuals* (*TPS Paradigm;*^6^ for introductory overviews, see Uher, 2015b, 2018a, pp. 3-8; Uher, 2021b, pp. 219–222; Uher, 2022b, pp. 3–6). This meta-paradigm was already applied, amongst others, to explore the epistemological and methodological fundamentals of data generation methods (Uher, 2018a, 2019, 2021a) and of theories and practices of measurement and pragmatic quantification across the sciences (Uher, 2020b, 2022a) as well as to scrutinise those underlying psychometrics and quantitative psychology (Uher, 2021c,d, 2022b, 2023a). Pertinent key problems were demonstrated empirically in multi-method comparisons (e.g., Uher et al., 2013a; Uher and Visalberghi, 2016; Uher et al., 2013b). The present article builds upon and substantially extends these previous analyses.

### 1.5 Outline of this article

This article offers a novel and ambitious transdisciplinary approach to advance the epistemological and methodological fundamentals of quantitative psychology by integrating relevant concepts from mathematical biophysics, metrology, linguistics, complexity science, psychology and philosophy of science. It elaborates the epistemic process structure of measurement, highlighting crucial differences to statistics (e.g., psychometrics). A focus is on elaborating the ways in which the peculiarities of language, when used in psychological methods (e.g., rating ‘scales', variables and models), obscure the epistemic differences between them. This confusion contributes to the common yet erroneous belief that statistics could constitute psychology's approach for ‘measuring' its study phenomena. The analyses are made with regard to psychology but equally apply to pertinent practices in other sciences.

Section 2 introduces fundamentals of measurement. These involve the measurement problem—the epistemically necessary distinction between the object of research and the objects used as measuring instruments as well as the conceptualisation of how the latter can provide information on the former. Measurement also requires the formal representation of observations in sign systems (e.g., data, formal models). The section presents Rosen's system of modelling relations as an abstract general model of the entire measurement process—from (1) conceptualising the objects of research, over (2) generating the data, (3) formally manipulating these data (e.g., statistical analysis) up to (4) interpreting the formal outcomes obtained with regard to the actual study phenomena. This process model is shown to underlie metrologists' approaches for tackling the problem of circularity in physical measurement, illustrated in the special cases of measurement coordination and calibration.

Section 3 applies these fundamentals to explore the challenges involved in establishing genuine analogues of measurement in psychology, which arise from the peculiarities of its study phenomena (e.g., higher-order complexity, non-ergodicity) and those of the language-based methods required for their exploration (e.g., inbuilt semantics). It demonstrates that psychology's focus on statistical modelling (e.g., psychometrics)—thus, on just one of the four necessary and interrelated modelling relations in Rosen's scheme—ignores the entire measurement process. But this often goes unnoticed because researchers consider only the general (dictionary) meanings of their verbal ‘scales'—their inbuilt semantics, yet ignore how raters actually interpret and use these ‘scales'. This introduces several breaks in the data's and model's relations to the actual phenomena that these are meant to represent. It also obscures psychology's measurement problem. This involves not just the crucial distinction between the phenomena studied (e.g., feelings) and those used as ‘instruments' for studying them (e.g., descriptions of feelings) but also individuals' (e.g., raters') local context-specific interactions with both. These complexify the ways in which epistemically justified (valid) information about the study phenomena can be obtained through language-based methods.

Section 4 shows that the frequent failure to distinguish the study phenomena from the means of their investigation (e.g., ‘instruments', formal models) confuses ontological with epistemological concepts—psychologists' cardinal error. This logical error is fuelled by quantitative psychologists' focus on statistics as well as by our human tendency to mistake verbal descriptions for the phenomena described. Many psychologists therefore mistake judgements of verbal statements for measurements of the phenomena described. Many also overlook that statistics can neither establish nor analyse a formal model's relations to the real phenomena studied. Establishing these relations requires genuine analogues of measurement for which the section elaborates necessary epistemological and methodological fundamentals. It closes by showing ways in which the powerful artificial intelligence systems (AI) now available for modelling human language can meaningfully support psychological research but also perpetuate psychologists' cardinal error.

## 2 Key problems of measurement

Measurement, in its most general sense, is a highly selective form of observation because ‘to measure' means that we must choose to measure *something* without having to measure *everything*. Every object of research may feature various non-equivalent properties (e.g., length, temperature and weight) as well as different quantitative entities of the same property (e.g., foot length, finger length and body height). Measurement is a process that involves the detection and recognition of selected properties in the object researched and that produces justified information about them (von Neumann, 1955; Uher, 2022b).

For simplicity, when ‘objects' are mentioned in the following, this is always meant to include their properties as well because we cannot measure objects in themselves (e.g., physical bodies) but only their specific properties (e.g., mass, voltage and temperature). Properties are also included when we understand by ‘objects of research' not just physical objects (e.g., individuals' bodies) but also non-physical phenomena (e.g., individuals' reasoning, beliefs and emotions)—thus, denoting the subject matter in general.

### 2.1 The measurement problem: Distinguishing the objects of research from the objects used as measuring instruments and conceptualising their interaction

We can describe all objects in their existence and being in the ‘world', thus ontologically. To describe how we can gain knowledge about a given object, thus epistemologically, we must distinguish the ontic object (the specific concrete entity) to be measured from the objects used for epistemic (knowledge-generating) purposes as measuring instruments. *Measurement* defines a theory-laden process structure that conceptualises the objects of research and the methods (including instruments) used to gain epistemically justified information about them (von Neumann, 1955).

Specifically, measurement requires an *empirical interaction* between the specific quantity to be measured (measurand) in the study object (e.g., the temperature of a cup of coffee) and the object used as instrument (e.g., mercury in glass tube). Measuring instruments must be designed such that they produce, through their empirical interactions with the measurand, distinctive *indications* that are observable for humans. In iterative processes of theorising and experimentation, scientists identify which variations of an instrument—when applied in defined ways (the *method*)—reliably produce distinct and for humans easily discernible patterns (e.g., linear extension of mercury in glass tubes). These indications are used to make inferences on the study object's specific state at the moment of interaction to obtain *information* about it. That is, scientists use their current state of knowledge to *decide* how to design specific objects as instruments, how to use them (methods) and which indications of their empirical interactions with the study object to consider as informative—thus, how ‘to read' these indications (Mari et al., 2021; Pattee, 2013; Tal, 2020; Uher, 2020b, 2023a).

In sum, the *measurement problem*^7^ concerns the epistemic distinction of the object of research from the objects used as measuring instrument. It requires their conceptualisation as well as that of their presumed empirical interaction under defined conditions (method) producing observable indications. To document and analyse them to derive measurement results, the observed and interpreted indications must be formally represented.

### 2.2 Measurement requires semiotic representation in rule-based formal models

The relations between physical properties are empirically given, invariant and lawful (those studied in metrology). But information about them can be *formalised* in various ways. Formalisms are conceptual, mathematical, algorithmic, representational and other abstract operations that follow logical, deductive or arbitrarily prescribed *rules*. In measurement, *formal representation* involves sign systems. Signs are composed of tokens (sign carriers; e.g., Latin or Greek letters, Arabic numerals) that are assigned meanings, which specify the information that these tokens are meant to represent (e.g., specific indications observed or quantitative relations). These sign systems constitute the *data* and formal models (e.g., variables, numerical values), which can be used to analyse the information represented (e.g., mathematically). The signs' meanings, however, because they just are as*sign*ed (attributed and ascribed), can vary. Numerals can represent numbers but also just order (e.g., door ‘numbers') or just nominal categories (e.g., genders). That is, formalisation is *arbitrary, non-physical and rule-based* (Abel, 2012; Pattee, 2013; Uher, 2023a; von Neumann, 1955).

In sum, semiotic (sign-based) representation is essential for all empirical sciences (Frigg and Nguyen, 2021; Pattee, 2013; van Fraassen, 2008). It requires that data and models are clearly distinguished from the objects that they semiotically represent. This separation is no philosophical doctrine but an epistemic necessity that follows from the *definition of a sign as something that stands for something other than itself* (Pattee, 2001; Peirce, 1958; Uher, 2020b, 2022b). The ways in which interpreted observations are encoded into data in a study are therefore crucial for understanding and analysing these data. The specific encoding is also essential for drawing justified conclusions from the analytical results about the actual objects explored. The study objects, their formal representations and the interrelations between both can be conceptualised and analysed in an overarching model.

### 2.3 The system of interrelated modelling relations underlying empirical science

Robert Rosen, a mathematical biophysicist and theoretical biologist, developed a general relational model to conceptualise the processes by which living beings selectively perceive specific parts of their environment and make sense of that information. Scientific knowledge generation is a special case of these fundamental processes. Rosen (1985, 1991, 1999) developed this process model mathematically building on earlier work by Rashevsky (1960b,a) and using category theory (Lennox, 2024).

#### 2.3.1 Category theory: Modelling the relations of relations between objects

Many psychologists associate mathematics solely with quantitative analysis (e.g., algebra, arithmetic, calculus). But mathematics also involves many non-quantitative branches, such as category theory, combinatorics, geometry, logic, set theory or topology (Linkov, 2024; Rudolph, 2013), which are also used in empirical sciences.

*Category theory* is a general mathematical theory to formally describe abstract structures and relations. In this theory, a category is a system of mathematical objects and their relations. The focus is on conceptualising these *relations, understood as morphisms, arrows or functors*, that map a source object to its target object in specific ways (e.g., through structure-preserving transformations). Category theory also permits to map these relations in themselves—thus, to map the relations between categories, termed *natural transformations*. Hence, category theory is about modelling (mathematical) objects, relations of objects as well as relations of relations (Leinster, 2014). This makes it suitable to model also the process of scientific modelling in itself (Rosen, 1985).

#### 2.3.2 Scientific modelling: Modelling the relations between causality, encoding, analysis and decoding

**Figure 1:**
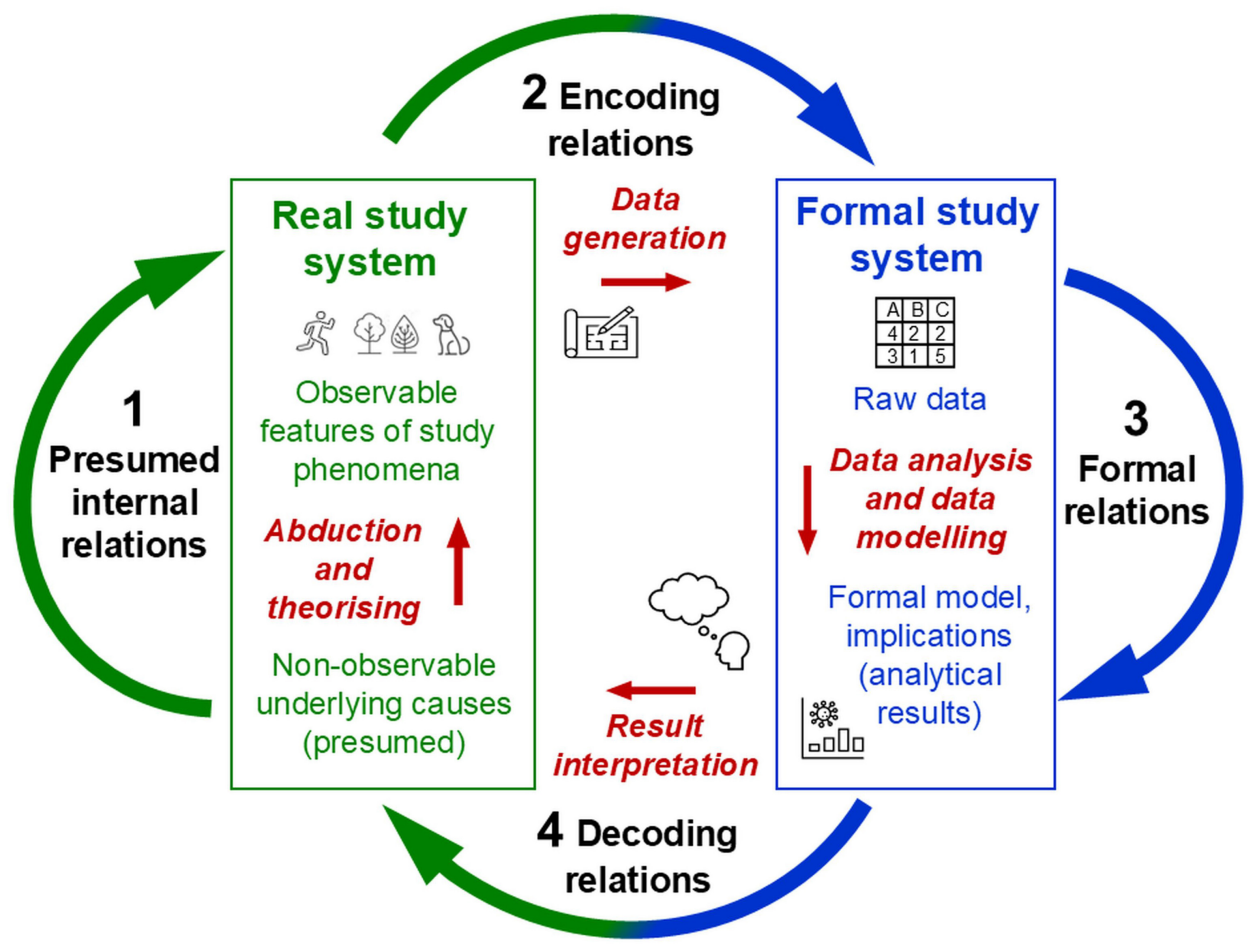
Rosen's general process structure of empirical science: A coherent system of four interrelated modelling relations. The real study system and the formal system used for studying it, conceptualised as mathematical objects, as well as the processes (mappings, relations) each within and back and forth between them, depicted as arrows. Adapted from Rosen (1985) and Uher (2022b, Figure 6).

For scientific inquiry in general, Rosen's system^8^ of interrelated modelling relations conceptualises the basic set of processes that are used to explore a specific part of the ‘world', conceived as the real system under study (object of research, study phenomena). These processes specify the ways in which this *real system being studied is mapped to the formal system that is used for studying it*. Stated in category-theoretic terms, these modelling relations relate disjoint categories of objects (Mikulecky, 2000, 2001). In everyday life, we intuitively establish such modelling relations whenever we try to make sense of the complex phenomena that we encounter, grounded in the general belief that these are not completely random but show some kind of order. Figure 1 illustrates the system of interrelated modelling relations, comprising the real study system and the formal system used for studying it as mathematical objects as well as the processes (mappings, relations) that are conceptualised within and between them, depicted as arrows. What do these different processes involve?

In science (and everyday life), when we perceive events as changes (e.g., in behaviour), we attribute to those changes some causes that we seek to explain (e.g., mental abilities, intentions) as possible causes of the observed events (e.g., through abduction; Peirce, 1958, CP 7.218). This (presumed) causal relation in the real system (e.g., a person) is depicted as arrow 1. Its exploration requires the *encoding* of the real changes observed. That is, selected indications that we deem relevant for exploring the presumed causal relations are encoded into objects and relations in the formal study system. These encoding relations^9^—the *data generation*—are depicted as arrow 2. The formal system is the explicit scientific model (or, in everyday life, the intuitive mental model) that we create to deal with the information obtained from our selected observations. It serves as a *surrogate* system that we can explore in ways that are not possible with the real system itself, such as mathematical analysis rather than physical dissection. Hence, the model is analysed *in lieu of* the actual objects of research (Rosen, 1985, 1991; Uher, 2015b,c,d).

We can manipulate the information encoded in the formal system in various ways using data modelling techniques (e.g., statistical or algorithmic analysis) to try to imitate the causal events that presumably occur in the real system (e.g., simulation models). Therefore, we must use our current knowledge of that real system (e.g., a person), its observable indications (e.g., behavioural responses) and (possible) non-observable internal relations (e.g., mental abilities) to decide which specific operational manipulations (e.g., statistical analysis) are appropriate to explore the information about that real system. Through manipulative changes and operations performed in the formal system—the *data analysis*—depicted as arrow 3, we obtain an *implication*, such as statistical or simulation results.

Once we believe that our formal system (e.g., structural equation model) is appropriate and may correspond to the presumed causal events in the real system, we must relate the results obtained in the formal system back to the real system studied. This *decoding* relation, depicted as arrow 4, requires interpreting the *formal* results with regard to the *non-formal* events occurring in the real study system. The aim is to check how well the formal model may represent the causes that we presume and that could explain the changes observed in that real system. Thus, decoding involves a mapping relation between disjoint categories of objects—thus, between the outcomes generated in a *formal* study system (e.g., mathematical) and the outcomes observable in a *real* study system (e.g., behavioural).

If the processes of encoding (2), implication (3) and decoding (4) appears to reproduce the presumed causal processes (1) sufficiently accurately, the system of modelling relations it said to *commute*. Commutation implies that the formal study system established in this process constitutes a successful model of the real system studied—expressed in category-theoretic terms by the equation: 1 = 2 + 3 + 4. Note that these numerals represent not numbers but different kinds of mapping relations, depicted as the four arrows in Figure 1. Hence, the system of modelling relations *conceptualises the relations between relations between objects* of different kinds (Rosen, 1985, 1991, 1999).

Rosen's process model is not commonly taught. Many scientists are even puzzled when they first encounter it (Mikulecky, 2011). This is astonishing and unfortunate because it conceptualises how empirical science, in general, and measurement, in particular, are done.

#### 2.3.3 How empirical science is done: The epistemic necessity of making subjective decisions

Rosen's system of interrelated modelling relations highlights several key points that are fundamental to empirical inquiry but often not well considered. First, it specifies that the system studied and the surrogate system (model) used for studying it are of different kinds—*real vs. formal*. The relations (mappings) established between them—encoding (arrow 2 in Figure 1) and decoding (arrow 4)—therefore involve transformations that cannot be derived from within either system. These relations are thus independent of both systems.

Specifically, potentially unlimited amounts of observations that can be made of a real study system must be mapped onto the limited sign system that is used as its formal model. Encoding therefore requires that scientists reduce and simplify their observations to only those elements that they interpret as relevant for their given research question and that they choose to encode as data. Thus, the essence of encoding is *high selectivity* and *reduction*. This requires *representational decisions* about what to represent, and what not, and about how to represent it (Harvard and Winsberg, 2022). For example, observations of variable and highly dynamic phenomena, such as behaviours (e.g., hand gestures), often require their encoding in fuzzy categories. This involves the mapping of fuzzy subsets of observations (e.g., physical states of fingers) into the same formal category (e.g., hand configurations; Allevard et al., 2005). That is, scientific representation, in general, and measurement, in particular, involves the *selective reductive mapping of an open domain of a study system to a closed sign system used as its surrogate model* (for general principles, see Uher, 2019).

Decoding—the inverse relation from the formal system back to the real system (arrow 4)—as well, is a delicate process that is prone to many potential points of failure. This is because it involves the transformation of results obtained through *formal* manipulations (e.g., mathematical, statistical), which are not possible in the *real* system (e.g., behavioural, psychical) itself (Mikulecky, 2000; Rosen, 1985, 1999). This epistemic necessity makes the modelling process prone to *methodomorphism*, whereby methods impose structures onto the results that, if erroneously attributed to the study phenomena, may (unintentionally) influence and limit the concepts and theories developed about them (Danziger, 1985; Uher, 2022b).

Second, Rosen's process model highlights that the only part of our scientific models that—taken by itself—is free from operational subjectivity is the formal study system (e.g., statistical model) that is used as a surrogate for the real system studied (arrow 3). However, the formal model is established by the scientists' choice and decisions and is therefore subjective in many ways as well (Mikulecky, 2000, 2011; Rosen, 1991; Strauch, 1976).

“This makes modelling as much an *art* as it is a part of science. Unfortunately, this is probably one of the least well appreciated aspects of the manner in which science is actually practised and, therefore, one which is often actively denied” (Mikulecky, 2000, p. 421).

In sum, Rosen's general model conceptualises the processes of empirical science that epistemically justify the representation of observable regularities by means of abstract (e.g., mathematical) models. These processes concern the *coordination (or correspondence) between theory and observable phenomena*, such as the applicability of theoretical concepts to concrete events—known as the *problem of coordination (or correspondence)* in science (Hempel, 1952; Margenau, 1950; Torgerson, 1958). To specify the conditions under which abstract representations can be applied to observable phenomena and used to investigate—and also to quantify—entities of non-observable phenomena, it requires measurement.

### 2.4 Tackling the epistemic circularity of measurement requires a coherent system of modelling relations

Any method of data generation involves categorisation, which enables basic forms of analysis, such as grouping or classifying objects by their similarities and differences. Measurement has advantages over mere categorisation^10^ by enabling more sophisticated analyses of categorised objects and their relations by additionally enabling the descriptive differentiation *between instances that are of the same kind (quality) and divisible—thus, that differ in quantity* (see Hartmann, 1964; Uher, 2018a, 2020b).

Key problems of measurement arise from the fact that many objects of research are not directly observable with our senses (e.g., electric potential, others' mental processes) or not accurately enough (e.g., weight of smaller objects). Rosen's process model underlies the approaches that are used to tackle these epistemic challenges, as illustrated here in the problems of measurement coordination and calibration.

#### 2.4.1 Measurement coordination: Exploring the relations between observable indications and unobservable measurands

*Measurement coordination* is the specific problem of how to justify the assumption that a specific measurement procedure does indeed allow us to measure a specific property in the absence of independent methods for measuring it. This involves the problem of how to justify that specific quantity values are assigned to specific measurands under a specific methodical procedure. Measurement coordination (also “problem of nomic measurement”; Chang, 2004) thus concerns the relations between the abstract terms used to express information about quantities and the ways of measuring those quantities (Luchetti, 2020).

Challenges arise from many phenomena's non-observability. We can often directly observe neither the specific quantity to be measured (measurand; e.g., a body's temperature) nor its relation to the observable quantitative indications that are produced by its interaction with the measuring instrument (e.g., length of mercury in glass tubes) and that may be useful to infer the measurand's unknown quantity. Thus, in the early stages of scientific inquiry, the mapping relation between indications and measurands is unknown (e.g., the function relating the values of length of mercury with temperature). But it cannot be determined empirically without already established, independent measurement methods—because it is through measurement that such relations are first established. This requires scientists to make preliminary decisions about what counts as an indication of the property studied (e.g., temperature)—not knowing their specific relations, nor (initially) what exactly that property actually is, nor what other factors may influence an instrument's observable indications.

The fact that these questions cannot be addressed independently of each other involves *epistemic circularity*, discussed in many sciences and philosophy for a century already (Chang, 2004; Luchetti, 2024; Mach, 1986; Reichenbach, 1920; van Fraassen, 2008). To tackle this problem, scientists must establish appropriate and independent sources of justification for a specific measurement procedure and the assignment of specific values to specific quantities of a specific property. To achieve this, they must coordinate several modelling relations and establish their interrelations coherently.

To construct thermometers, for example, scientists began with preliminary definitions that coordinated a preliminary theoretical concept of temperature with empirical indications that could be obtained from preliminary instruments and their variations. They filled various liquids or gases in glass tubes and studied variations in their extension (volume) obtained from various heat-producing operations. Presuming a linear invariant relation between volume and temperature, scientists experimented with different substances (e.g., alcohol, hydrogen, mercury and water) to identify under which standardised conditions (e.g., pressure and heat production) which substance reliably produces distinct (e.g., monotonously increasing) indications, thus showing thermometric properties. From consistent indications produced by different thermometric substances, scientists could develop different kinds of thermometers, thus enabling triangulation. The redefinition of temperature as the average kinetic energy of particles provided a theoretical foundation to substantiate the linear invariant relation between temperature and the volume of specific substances used in thermometers (under specified conditions; Chang, 2004; JCGM100:2008, 2008; Kellen et al., 2021; Uher, 2020b).

The problem of measurement coordination and its inevitable epistemic circularity can thus be tackled through iterative processes in which a coherent system of assumptions is established to justify specific knowledge claims—using a *coherentist approach* (Olsson, 2023). With each epistemic iteration, the theoretical concept is re-coordinated to more reliable indications, which in turn enables more precise tests of predictions, more advanced theories, more refined and more standardised methods and instruments of measurement, and so on (Luchetti, 2024; Tal, 2020; van Fraassen, 2008). Through these *iterative feedback loops*, scientists systematically develop epistemic justifications for having implemented *coordinated connections* between the (presumed) non-observable measurand (e.g., a cup of coffee's specific temperature), the observable indications produced by its lawful (invariant) interaction with the measuring instrument (e.g., length of mercury), a known reference quantity (e.g., another thermometer used for calibration), and the semiotic representations of the information thus-obtained (e.g., ‘37°Celsius', ‘98.6° Fahrenheit'). This information is then mathematically analysed in the formal system. The obtained result can be used to make justified inferences on the specific quantity of the non-observable measurand.

**Figure 2:**
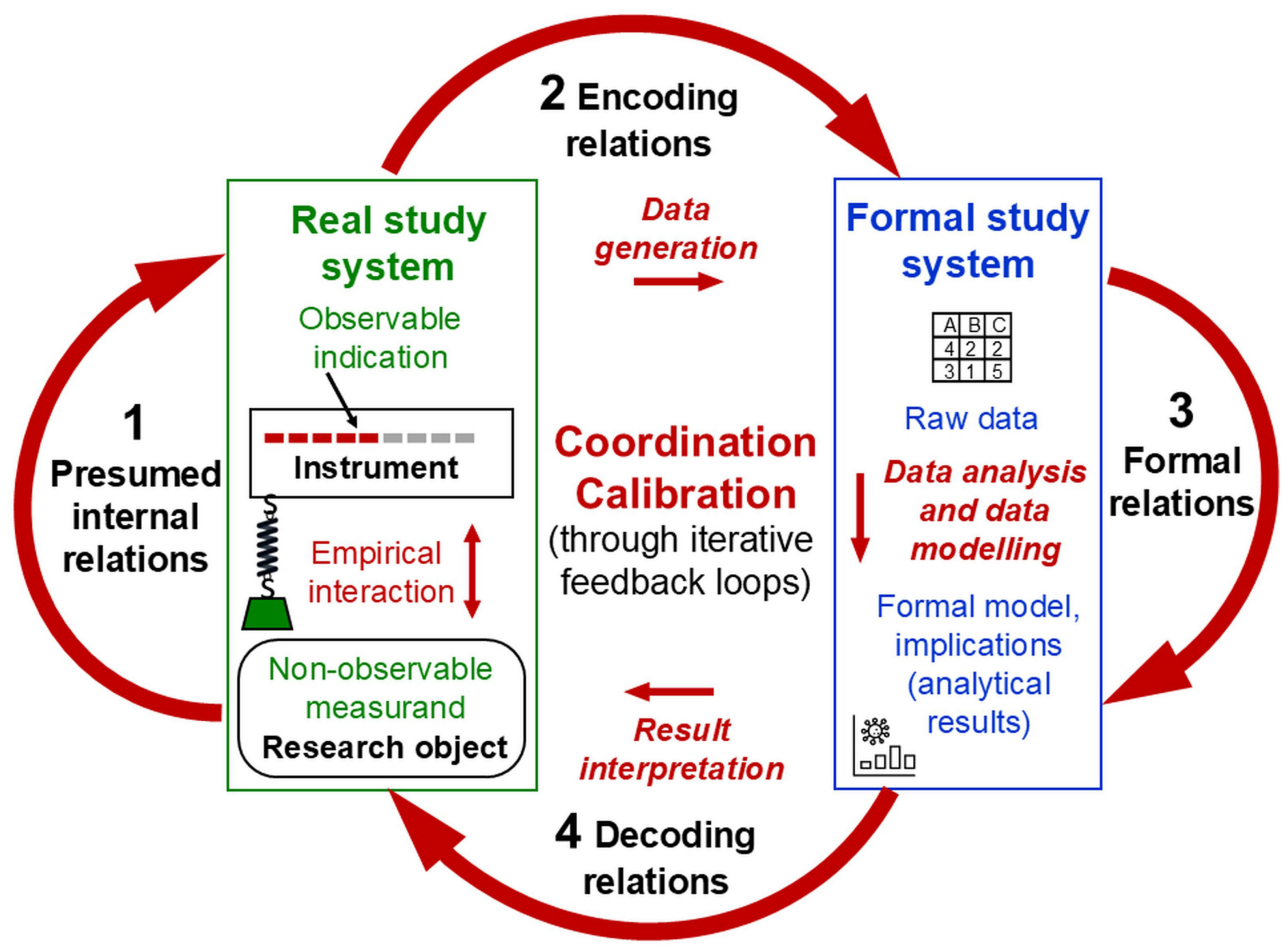
Physical measurement: A coherent system of four coordinated and calibrated modelling relations.

Rosen's general model allows for conceptualising the process structure underlying measurement coordination. Accordingly, this involves modelling the presumed relations within the real study system, comprising the non-observable object of research (measurand), the object used as instruments and the observable indication produced from their (non-observable) empirical interaction. Their presumed causal relations (arrow 1 in Figure 2) are then explored empirically through *unbroken documented traceable* relations to, within and back from the formal system that is used to study that real system (arrows 2, 3 and 4). In iterative feedback loops, the four modelling relations in Rosen's system (arrows 1 to 4) are passed through over and over again, thereby *re-coordinating* them with one another until their commutativity is established, indicating successful modelling of the real study system.

Necessarily, scientists can start to establish measurement coordination only from preliminary assumptions and theories about the study property and from preliminary instruments, methods and decisions on arbitrary encoding rules to obtain first empirical data. They must use preliminary, yet theoretically informed, analytical operations to obtain possibly informative implications. When decoding and interpreting these analytical results, scientists can also make only preliminary assumptions about the implications that these may have for the presumed relations between instrument indications and measurand. Each iteration in the overarching model of a measurement process enables new theoretical, methodical and empirical insights and refinements, which mutually stimulate each other, leading to cascades of development through which a coherent system of epistemically justified knowledge claims is established.

These iterative processes also involve testing and adjusting the specific parameters of a given measurement procedure—through calibration.

#### 2.4.2 Calibration: Modelling precision and uncertainty in measurement

*Calibration* procedures establish reliable relations between the instrument indications obtained under a given method in the real study system and the measurement results obtained in the formal model, which specify information about the actual (non-observable) quantity to be measured (measurand). Calibration is theoretically constructed and empirically tested by modelling uncertainties and systematic errors under idealised theoretical and statistical assumptions (e.g., about distribution patterns and the randomness of influencing factors). The aim is to improve the accuracy of the measurement results by specifying the ranges of uncertainties and errors for all parameters involved in a given measurement procedure. This allows for incorporating corrections for systematic effects (e.g., of pressure on temperature) and for adjusting inconsistent observations of instrument indications (Chang, 2004; Luchetti, 2020; McClimans et al., 2017; Tal, 2017).

That is, calibration involves modelling activities that are aimed at refining the coordinated structure of a measurement process. In Rosen's scheme, this means that the parameters used to establish proportional (quantitative) relations in the measurement model are adjusted within and across all four modelling relations (arrows 1 to 4 in Figure 2). These modelling relations are passed through in iterative feedback loops to obtain quantitative parameter value ranges that maximise the predictive accuracy of the overarching model. Thus, *calibration* refers to the coordination of abstract quantity terms in the formal model with the specific quantities to be measured in real study objects when a specific measurement method (including measuring instrument) is used (Luchetti, 2020; McClimans et al., 2017).

This model-based view of calibration illustrates the coherentist approach that is necessary to tackle the epistemic circularity of measurement. This involves establishing theoretical and empirical justifications for the assumption that a specific method (including instrument) enables the measurement of a specific property in absence of other independent methods for measuring it. Once different methods (and instruments) for measuring the same property (e.g., temperature) are developed, uncertainties and systematic errors can also be modelled across different procedures and instruments, such as to calibrate thermometers involving different kinds of thermometric substances (e.g., gases and fluids; Chang, 2004).

Calibration processes are necessary to implement numerical traceability^11^—thus, to establish for the numerical values used as measurement results a publicly interpretable meaning regarding the specific quantities measured (*how* much of the studied property that is; Uher, 2022a). To ensure that measurement results are reliably interpretable and represent the same quantitative information regarding the measurands across time and contexts (e.g., specific weight of 1 kilogram), metrologists defined primary references, which are internationally accepted (e.g., through legislation) and assumed to be stable (e.g., prototype kilogramme^12^). From each primary reference, large networks of unbroken documented connection chains were established (via national references) to all working references that are used in measurement procedures in research and everyday life (e.g., laboratory weighing scales, household thermometers; JCGM200:2012, 2012). These *calibration chains* specify uncertainties and errors as quantitative indications of the quality of a measurement result to assess its precision and accuracy (JCGM100:2008, 2008; Uher, 2020b).

#### 2.4.3 The theoretical and empirical process structure of measurement: A coordinated and calibrated system of four interrelated modelling relations

The essence of measurement is thus a theory-laden process structure that involves modelling relations each within a real and a formal study system as well as back and forth between them, which are coherently connected with one another in an overarching process, as conceptualised in Rosen's general model (Figure 2). This requires data generation methods that enable empirical interactions of the non-observable quantities to be measured with a measuring instrument. Identifying observable indications of these interactions that are (possibly) informative about these measurands requires a general model of coherent and epistemically justified interrelations within and between the real and the formal study system. These are *re-*coordinated and *re-*calibrated with one another by empirically *re-*testing the presumed relations (e.g., comparing predicted and observed indications), *re-*adjusting their parameters (e.g., errors, uncertainties) and *re*-fining assumptions (e.g., randomness).

In sum, a coordinated and calibrated system of interrelated modelling activities is necessary to empirically implement unbroken traceable connection chains that establish proportional (quantitative) relations between the measurement results obtained in the formal model and both (1) the measurand's unknown quantity (data generation traceability) and (2) a known reference quantity (numerical traceability) in the real study system. Measurement models thus-developed allow us to derive from defined observable instrument indications calibrated measurement results that can be (1) justifiably attributed to the measurands, and (2) publicly interpreted in their quantitative meaning regarding those measurands—the two epistemic criteria of measurement across sciences (Uher, 2020b, 2022a). The insights gained from iteratively developing the process structure of a measurement model may also necessitate a revision of the definitions and theoretical explanations of the objects and relations in the real system (e.g., temperature redefined as average kinetic particle energy).

Clearly, physical measurement procedures cannot be directly applied to psychology. But what specifically are the challenges for devising *analogous* processes in psychology?

## 3 Psychology's inherent challenges for quantitative research

The history of metrology testifies to the challenges involved in tackling the problems of measurement coordination and calibration in physical measurement (Chang, 2004)—thus, in the study of invariant relations in non-living nature, which can therefore be formalised in immutable laws, natural constants and mathematical formulas. Psychology, however, explores phenomena (e.g., behaviours, thoughts and beliefs) that are—*in themselves*—variable, context-dependent, changing and developing over time (Uher, 2021b). Such peculiarities are characteristic of living systems (e.g., psyche and society) and not studied in metrology. These peculiarities entail that the low replicability of psychological findings is not just an epistemic problem that could be remedied with more transparent and robust methods, as many currently believe. Rather, it is also a reflection of the indeterminate variability and changeability of the study phenomena themselves (arrow 1 in Figure 1). Low replicability of psychological findings thus reflects not just epistemic uncertainty of ‘measurement' but also *fundamental ontic indetermination* (Scholz, 2024).

### 3.1 Psychology's study phenomena: Peculiarities of higher-order complexity

Living systems (e.g., biotic, psychical and social) are of higher order complexity. They feature peculiarities not known from non-living systems (Baianu and Poli, 2011; Morin, 2008).

#### 3.1.1 Emergent properties not present in the processes from which they arise

In higher-order (super) complex systems, interactions occur between various kinds of processes on different levels of organisation from which novel properties emerge on the level of their whole that are not present in the single processes from which they arise. These novel, higher-level properties can also feed back to and change the lower-level processes from which they emerge. Such dynamic multi-level feedback loops lead to continuous change and irreversible development on all levels of organisation (Morin, 1992; Rosen, 1970, 1999).

Human languages, for example, gradually emerged from individuals' interactions with one another. The language of a community, in turn, mediates and shapes the ways in which its single individuals perceive, think and organise their experiences into abstract categories. Through dynamic multi-level feedback processes over time, individuals, their community and their language mutually influence each other, thereby developing continuously further and getting ever more complex (Boroditsky, 2018; Deutscher, 2006; Valsiner, 2007; Vygotsky, 1962). This entanglement of mind and language first enables the use of language-based methods in science. But the intricacies of language also promote conceptual confusions, which are still largely overlooked, as this section will show.

Emergence also entails complex relations between the levels of parts and wholes.

#### 3.1.2 Complex wholes and their parts: One–to–many, many–to–one and many–to–many relations

In living systems (e.g., individuals), the same process (e.g., a specific feeling) can generate different outcomes (e.g., different behaviours) in different times, contexts or individuals—thus, involving *one–to–many relations* (multifinality, pluripotency). Vice versa, different processes (e.g., of abstract thinking) can generate the same outcome (e.g., solving the same task)—thus, involving *many–to–one relations* (equifinality, degeneracy; Cicchetti and Rogosch, 1996; Mason, 2010; Richters, 2021; Sato et al., 2009; Toomela, 2008; Uher, 2022b). To consider multiple processes and outcomes at once, we must conceptualise *many–to–many relations* between the parts and their whole on different levels of organisation.

This entails that specific relations from observable indications to non-observable phenomena that apply *to all individuals in all contexts and all times* cannot be identified. This complicates the possibilities for solving the problem of *measurement coordination in psychology*. Specifically, complex relations challenge the appropriateness of the sample-level statistics commonly used in psychology, which are aimed at identifying invariant^13^ (e.g., cause–effect) relations, such as between latent and manifest variables in factor analyses or structural equation models—that is, one–to–one relations.

Complex multi-level relations also entail the fact that the properties of parts identified in isolation (e.g., cells) cannot explain the whole (e.g., organism) because its properties emerge only from the parts' *joint* interactions. Changes in single parts or single relations between them can change the properties of the whole. Psychical processes cannot even be isolated from one another, although they can be qualitatively distinguished (Luria, 1966). Thus, *complex wholes are more than and different from the sum of their parts* (Morin, 1992, 2008; Nowotny, 2005; Ramage and Shipp, 2020; Uher, 2024). All this entails that living systems cannot be explored by reducing them to the parts of which they are composed (e.g., organisms to cells), as this is possible for the non-living systems (e.g., technical) featuring invariant relations as studied in metrology (Rosen, 1985, 1991).

#### 3.1.3 Humans are thinking intentional agents who make sense of their ‘world'

Psychologists also cannot ignore the fact that humans are thinking agents who have aims, goals and values that they pursue with intention and who can anticipate (mentally model) future outcomes and proactively adjust their actions accordingly. Humans hold personal (subjective) and socio-cultural views on their ‘world', including on the psychological studies in which they partake. Individuals memorise and learn. Therefore, simple repetitions of identical study conditions (e.g., experiments and items) cannot be used (Danziger, 1990; Kelly, 1955; Shweder, 1977; Smedslund, 2016a; Uher, 2015a; Valsiner, 1998).

In sum, psychology's study phenomena feature peculiarities that do not occur in the properties amenable to physical measurement. These peculiarities complicate the design of analogous research processes that meet the two epistemic criteria of measurement. In the following, we explore these complications stepwise, starting with the level of data analyses.

### 3.2 Psychology's focus on aggregate level analysis

Psychology's primary scientific focus (unlike sociology's) is on the *individual*, which constitutes its *theoretical unit* of analysis. The *empirical units* of analysis in psychological ‘measurement', however, are *groups*. Why is that so? And what justifies the assumption that results obtained on aggregate levels are suited to quantify individual level phenomena?

#### 3.2.1 Indefinitely complex and uncontrollable influence factors: Randomisation and large sample analyses

Unlike metrologists and physical scientists, psychologists cannot isolate their study objects and experimentally manipulate the (presumed) quantities to be measured in them, such as individuals' processing speed, reasoning abilities or beliefs (Trendler, 2009). Moreover, in physical measurement, influencing factors involve comparably few and exclusively here-and-now factors. By contrast, the factors influencing psychology's study phenomena, such as internal and external conditions causing mental distraction, are indefinitely complex and ever-changing and can even transcend the here-and-now (Barrett et al., 2010; Smedslund et al., 2022; Uher, 2016a).

To deal with these challenges, psychologists study groups of individuals that are assumed to be sampled randomly with regard to these unspecifiable and uncontrollable influence factors. To estimate the impact of these factors, psychologists analyse samples that are large enough to allow for identifying regularities beyond pure randomness in the study phenomena (e.g., by comparing experimental with control groups). This approach necessitates the statistical analysis of group-level distribution patterns. The statistical results, however, are commonly interpreted with regard to the single individuals (e.g., their beliefs). That is, from statistical analysis to result interpretation, psychologists shift their unit of analysis from the sample back again to the individual—without explanation but in line with their theoretical unit of analysis (Danziger, 1985; Richters, 2021; Uher, 2022b).

But in what ways can results on aggregates be informative about single individuals?

#### 3.2.2 The ergodic fallacy: Psychology's common sample–to–individual inferences built on mathematical errors

Statistical analyses of aggregated data sets can reveal information about the single cases only when their synchronic and diachronic variations are equal (isomorphic)—a property of some stochastic and dynamic processes in non-living systems termed *ergodicity*. In the 1930s already, mathematical-statistical (ergodic) theorems^14^ were used to prove that ergodicity does not hold for cases that vary, change and develop (Birkhoff, 1931). Hence, *psychology's study phenomena are non-ergodic*, which means that between-individual (synchronic) variations are uninformative about within-individual (diachronic) variations. Thus, when using sample-level analyses (e.g., factor analysis) to study individual-level phenomena (e.g., psychical ‘mechanisms'), psychologists commit an inferential error—the *ergodic fallacy* (Bergman and Trost, 2006; Danziger, 1990; Lamiell, 2018, 2019; Molenaar and Campbell, 2009; Richters, 2021; Smedslund, 2016a, 2021; Speelman and McGann, 2020; Uher, 2022b, 2015d; Valsiner, 2014b; van Geert, 2011; von Eye and Bogat, 2006).

In sum, the higher-order complexity of psychology's study phenomena poses considerable challenges for empirical research. The uncontrollability of influencing factors requires statistical analyses of large samples. But individuals' complexity renders sample-level results uninformative about the single individual. These and further problems complicate the development of genuine analogues of measurement.

### 3.3 Psychological ‘measurement' theories: Failure to conceptualise a coherent system of interrelated modelling relations

As Section 2 showed, measurement requires a coherent system of four interrelated modelling relations—each within a real and a formal study system and back and forth between them (arrows 1 to 4 in Figure 2). The ‘measurement' theories established in psychology, however, such as Representational Theory of Measurement (RTM) and psychometrics, focus on just some of these modelling relations, thereby ignoring the overall model that is necessary to relate them coherently to one another.

#### 3.3.1 Representational Theory of Measurement: Simple observable relations represented in mathematical relations

Representational Theory of Measurement (RTM; Krantz et al., 1971; Luce et al., 1990; Suppes et al., 1989) formalises axiomatic conditions by which observable relational structures can be mapped onto symbolic relational structures. It provides mathematical theories for this mapping (*representation theorem*), including permissible operations for transforming the symbolic structures without breaking their mapping relations onto the observable structures (*uniqueness theorem*; Narens, 2002; Vessonen, 2017). That is, representational theory specifies the semiotic representation of observable indications—the encoding and decoding relations in Rosen's structural model. The theory's focus on isomorphisms—thus, on reversible one–to–one relations between observables and data (arrows 2 and 4 in Figure 2)—presupposes that the objects of research feature properties with quantitative relations that are directly observable (e.g., ‘greater than' or ‘less than'). Such relations can be mapped straightforwardly onto a symbolic system that preserves these relations (e.g., ordinal variables; Suppes and Zinnes, 1963).

Psychologists, however, encounter tremendous challenges when trying to identify empirical regularities in observable (presumed) indications of psychical phenomena as well as (possibly) quantitative relations in these indications (e.g., in behaviours, performances). Highly variable dynamic study phenomena necessitate fuzzy encoding relations, which can be defined and established differently. Specifying such many–to–one encoding relations is seldom straightforward. It requires theory-driven (in parts also arbitrary) decisions of what to formally represent and how. These decisions may impact the information encoded in the data—and thus, the results that can be obtained from them (Uher, 2019). All this further complicates the problem of measurement coordination in psychology (Luchetti, 2024; Uher, 2022b, 2023a). In all sciences, measurement requires highly selective and reductive representation. In psychology, it requires mapping information about a highly complex study system, which cannot be fully defined in principle (e.g., behavioural, psychical and belief systems), to a simple system, which can be fully defined (e.g., structural equation model).

Representational theory, however, provides neither concepts nor procedures for how and why some observations should be mapped to a symbolic relational system (Mari et al., 2017; Schwager, 1991). Concretely, it provides no concepts to specify the relations between observables and the (non-observable) quantity to be measured (measurand) in a study object. Nor does it provide concepts to specify the measurand's empirical interactions with the measuring instrument that first produce these observable indications (arrow 1 in Figure 2). Such specifications, however, are necessary to design suitable instruments and to operate them in defined empirical procedures (methods). They are also necessary to justify why some indications, but not others, should be observed—thus, to generate data that can be informative about the measurands (arrow 2). In view of this, it is unsurprising that representational theory provides no concepts for controlling the effects of influence properties and for modelling precision and uncertainty either. The theory confines empirical research to just simple observables that can be mapped easily onto useful mathematical relations, and vice versa. As Rosen (1985) highlighted, however, encoding and decoding (arrows 2 and 4) relations involve transformations that cannot be derived from within either system and that are therefore independent of these systems.

In sum, representational theory ignores the entire system of traceable modelling relations that must be coordinated and calibrated with one another to enable epistemically justified and publicly interpretable inferences from defined observable indications to the (non-observable) quantity of interest—the key criteria of measurement (Figure 2). Instead, it stipulates a purely representationalist and operationalist procedure that simplifies observations such as to align them to mathematically useful relations—in line with Stevens (1946, p. 667) earlier redefinition of ‘measurement' as “the assignment of numerals to objects according to a rule” (other than randomness; Stevens, 1957). These simplistic notions formed the basis for psychology's theories and practices of pragmatic quantification and separated them from those of measurement used in metrology and physics (Mari et al., 2021; McGrane, 2015; Uher, 2021c). Still today, these representationalist and operationalist notions of ‘measurement' underlie the psychology's main method of quantitative data generation—rating ‘scales', in which numerical scores are straightforwardly assigned to specific answer categories.

These representationalist and operationalist notions of ‘measurement' also underlie psychometrics—meant to mean the “science of measuring the mind” (Borsboom, 2005).

#### 3.3.2 Psychometrics: Formal modelling aligned to statistical criteria and theories, enabling pragmatic result-dependent data generation

**Figure 3:**
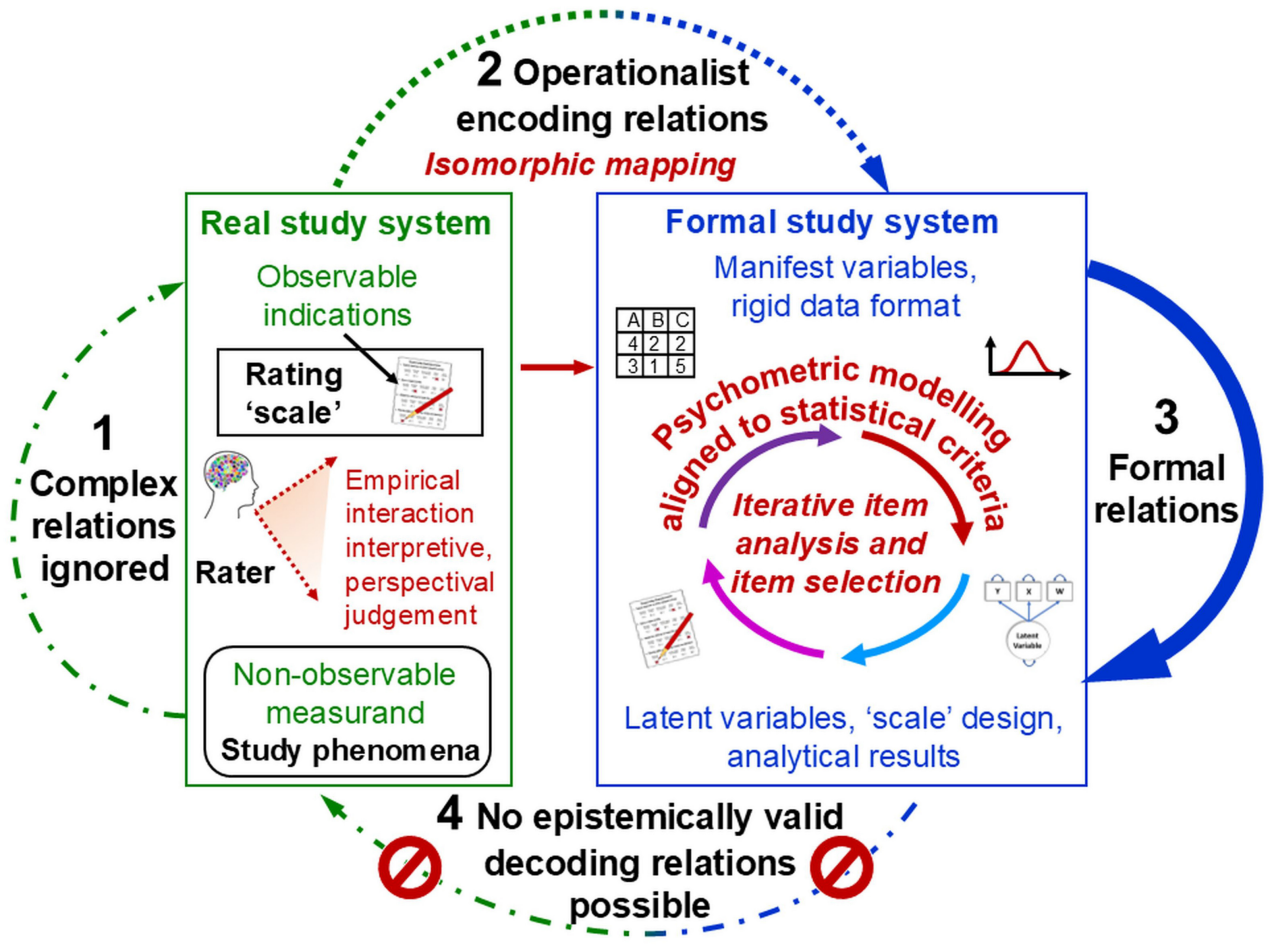
Psychometrics: Result-dependent methods of data generation and data analysis. Adapted from Uher (2022b, Figure 9).

The triviality of the isomorphic relations in encoding and decoding (arrows 2 and 4 in Figure 3)—stipulated by representational theory and Steven's redefinition of ‘measurement' and implemented in rating methods—shifted psychologists' focus away from the real study system (arrow 1) to the formal model (arrow 3). Statistical theories and methods, such as those of psychometrics, were advanced to develop sophisticated models and analyses that enable the reliable and purposeful discrimination between cases (e.g., individuals). This involved designing psychometric ‘instruments' that allow for generating data with useful statistical properties (e.g., normal distribution, high item discrimination). Stevens' (1946) mathematically defined ‘scales' (e.g., ordinal, interval, ratio)—although these are neither exhaustive nor universally accepted (Thomas, 2019; Uher, 2022a; Velleman and Wilkinson, 1993)—contributed further concepts to this end.

Psychometrics serves its pragmatic and utilitarian purposes well. But its approaches align the formal system (arrow 2 in Figure 3) to the criteria and theories on which the formal model and its manipulations are built (e.g., item-response theory)—regardless of the specific phenomena studied (e.g., behaviours and beliefs). Indeed, some even consider representation to be irrelevant for psychological ‘measurement' (e.g., Borsboom and Mellenbergh, 2004; Michell, 1999). The epistemic necessity to conceptualise and implement an empirical interaction with the (non-observable) quantity to be measured in individuals gets out of sight. Psychometricians also overlook that identifying observable indications of these empirical interactions that may be informative about the measurands requires theoretical knowledge about *both* the real system studied and the methodical system (including the ‘measuring instruments') used to study it (arrow 1). Instead, psychometricians choose ‘instrument' indications (e.g., answer categories on rating ‘scales') onto which pragmatically useful data structures (e.g., fixed numerical value ranges) can be mapped straightforwardly (Uher, 2018a, 2022a,b).

Hence, by focusing on statistical modelling (arrow 3, Figure 3), psychometricians neglect the three other modelling relations (arrows 1, 2 and 4) without which a formal system cannot be coordinated and calibrated with the real study system. Their interrelations are neither conceptualised nor empirically established but simply decreed, such as in the operationalist definition of ‘intelligence' as what an IQ-test measures (Boring, 1923; van der Maas et al., 2014). Specifically, psychometricians fail to conceptualise the real study system—comprising the study object, the measurand, the instrument and their empirical interaction producing observable indications. Therefore, they overlook that the quantitative scores recorded in ‘intelligence test' (e.g., number of correct answers) are properties of the *outcomes* of intellectual abilities but not of these abilities themselves.

Indeed, any test performance may involve several, qualitatively different intellectual abilities and modes of processing (e.g., symbolic, situational and verbal). More intelligent individuals may use *qualitatively* different (e.g., more efficient) abilities than less intelligent ones, different modes of processing and even multiple ones dynamically, leading to *quantitatively* different test performances. But none of these intricate many–to–one, one–to–many and many–to–many relations are considered in psychometrics. It only models relations of specific test outcomes to the abstract ‘intelligence' construct that they operationally define, which is then re-interpreted as a real unitary object to be ‘measured' (Khatin-Zadeh et al., 2025; Toomela, 2008; Uher, 2020b, 2021d,c, 2022b).

Psychometrics also provides neither concepts nor procedures for establishing unbroken traceable connections between results, measurands and instruments. As Section 2 showed, these are necessary to address the problems of circularity and coordination—thus, to provide evidence that a specific measurement procedure does indeed allow us to measure a specific property. Still, psychometric validity is often defined as “a property of measurement instruments that codes whether these instruments are sensitive to variation in a target attribute.” This is “broadly consistent with the view that a test is valid if it measures what it should measure” (Borsboom et al., 2009, p. 135). Such causal measurand–result relations, however, are neither conceptualised nor empirically implemented. Therefore, the validity of psychometric ‘instruments' can be analysed only regarding the coherence of their results with those obtained with other psychometric ‘instruments' that are targeted at study phenomena that are considered to be theoretically related (Cronbach and Meehl, 1955).

These inconsistencies reflect the confusion between two incompatible epistemological frameworks, which is intrinsic to psychometrics. Psychometricians' declared aims and result interpretations invoke the *realist framework* of measurement. But psychometric theories and the implemented empirical practices are built on a *pragmatist utilitarian framework*. These pragmatic fundamentals are reflected, however, in validity concepts that focus on the results' practical use, such as their social and ethical consequences (Messick, 1995), or the inferences and actions that can be derived from them, such as regarding their plausibility and appropriateness (Kane, 2013; Uher, 2021d,c).

In sum, psychometricians focus on the formal model and its analyses (arrow 3) but neglect conceptualising and empirically implementing its interrelations with the real study system (arrows 1, 2 and 4). This aligns psychometric methods (e.g., ‘scales') to statistical theories rather than to the study phenomena, thus enabling not traceable but *result-dependent data generation* (Figure 3) and leading to methodomorphism (Uher, 2020b, 2021c,d, 2022b, 2023a). This focus on statistical modelling abstracts away from the processes of measurement and thus, the actual study phenomena. It also obscures the data's meaning.

### 3.4 Psychology's focus on statistical modelling obscures the data's meaning

Psychologists' focus on statistics obscures the two distinct meanings that must always be conceptualised for empirical data—and thus, what these data actually represent. This highlights peculiarities of sign systems that are crucial for enabling empirical science.

#### 3.4.1 Statistics and algorithms: Analysing the syntax of data irrespective of their meaning with regard to the study phenomena—their empirical semantics

Statistics and other algorithms (e.g., data mining, machine learning) are formal methods that enable manipulations of formal models (arrow 3 in Figure 1), such as to identify regularities, interdependences, compatibilities or network structures in data sets. Statistical and algorithmic methods allow us to study how the data (e.g., variables, values) in a formal model are related to one another—their *syntax*. In linguistics, syntax denotes the set of language rules (e.g., grammar) that specify the structure and ordering in which words and phrases can be combined linearly to form sentences, which may influence their function in a sentence. Syntax allows us to indicate, for example, who is the actor of an activity and who the recipient. The words' meaning, in turn, arises from what they stand for and represent—their *semantics*. In linguistics, semantics denotes the set of rules that specify the meaning that words, phrases and sentences conventionally convey with regard to what they refer to (their referents). Thus, semantic meaning is established by way of a formal relation (Michaelis, 2003; Pattee, 2001).

The distinction between syntax and semantics is universal and basic to all life. In biophysics and biosemiotics, the DNA's *syntax* denotes the physical linear sequence of base pairs (copied into RNA through *transcription*^15^), whereas its *semantics* denotes the meaning that specific triplets on that sequence (codons) have for cells to instruct the production of specific amino acids (*translation*). That is, base triplets (codons) serve as physical tokens and carriers (“sign vehicles”) that stand for something else (amino acids). What specifically they stand for is determined not physically (not by their molecular structure) but formally—on the basis of rules (described in the codon table; Abel, 2009, 2012; Pattee, 2021).

**Figure 4:**
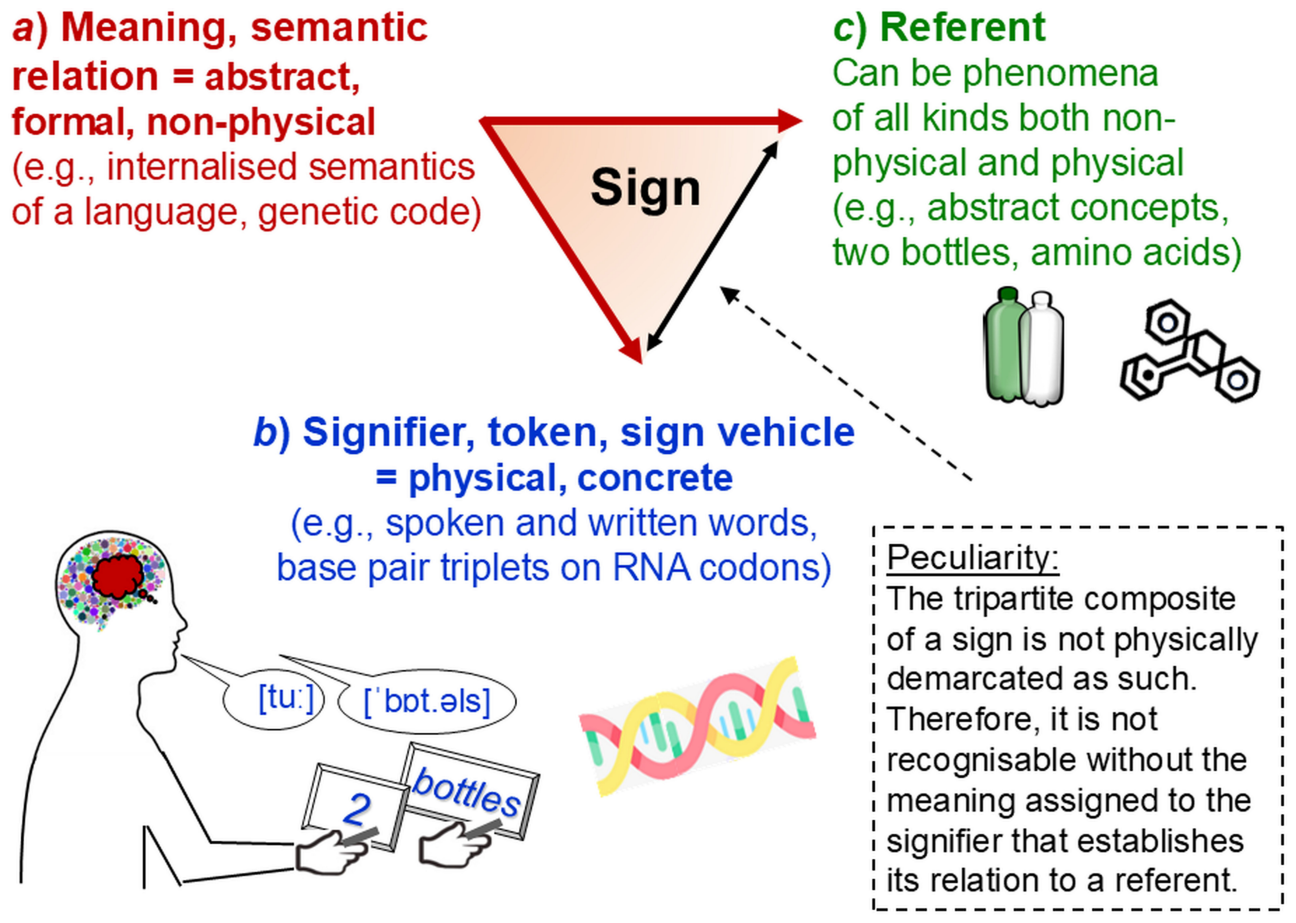
Signs: The meaning assigned to a signifier establishes its semantic relation to a referent. Adapted from Uher (2018a, Figure 3).

This illustrates the three distinct parts from which a *sign* emerges a whole (Figure 4). The *signifier* (e.g., a written word, an RNA codon) is the physical carrier that stands for something other than itself, which it represents, signifies or refers to—its *referent* (e.g., object, amino acid). The signifier's formal relation to a specific referent defines its semantic *meaning* (Ogden and Richards, 1923; Peirce, 1958; Rød, 2004; Uher, 2021c, 2022b; Vygotsky, 1962).

**Figure 5:**
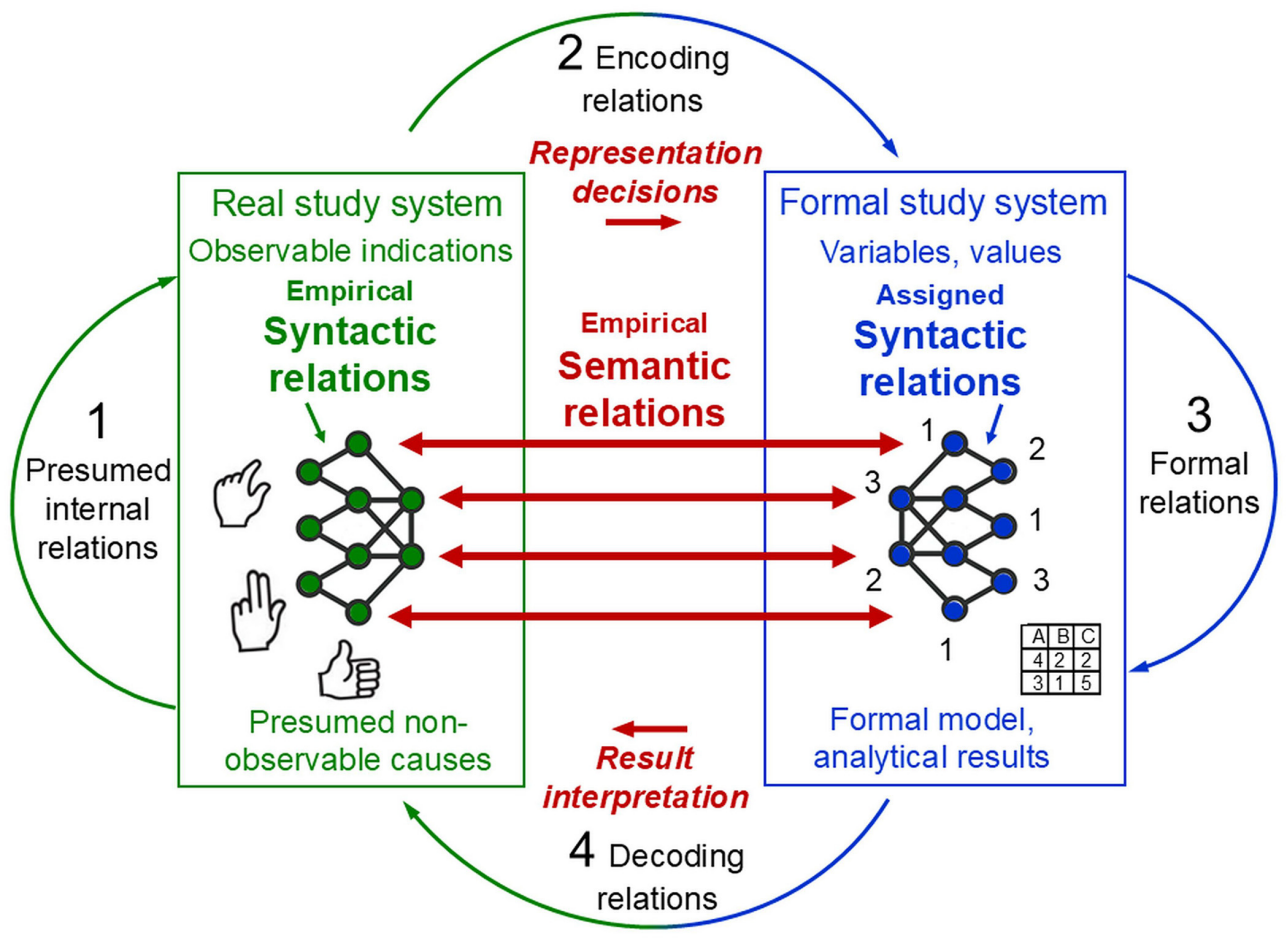
Data in formal models: Semantic and syntactic meanings.

Hence, for empirical studies, we must always as*sign* both a syntactic and a semantic meaning to the signifiers that we use as data (e.g., variable names, numerical values). The *syntactic meaning* defines the data's relations within the formal system (Figure 5). Nominal, ordinal and ratio variable meanings, for example, define different mathematical relations for the same numerical values ‘1', ‘2' and ‘3'. Thus, *syntactically*, these data may denote categorical (qualitative) differences, order relations or quantitative relations in a model. The empirically established *semantic meaning*, in turn, anchors the data in that selected part of ‘reality' that they are meant to represent and for which they serve as a surrogate to enable formal analyses (arrow 2). Thus, *semantically*, the same numerical data ‘1', ‘2' and ‘3' may refer, in different variables, to individuals' genders, shoe sizes, finger rings or hand gestures.

Statistics and other algorithms operate solely on the basis of a model's syntactic relations (arrow 3). They can neither establish nor analyse a model's relations to a real study system (e.g., genders, shoes or gestures). These methods perform purely syntactic data analyses *no matter what these data stand for* in a study—thus, regardless of their semantic relations to the real study system (arrows 2 and 4 in Figure 5). Ignoring the data's *empirical semantics* can lead to confusion about the syntactic relations that should be assigned to them (arrow 3) to appropriately match the empirical syntax of the real system (arrow 1).

#### 3.4.2 Ignoring the data's empirically established semantics can lead to inappropriate syntactic (statistical) analyses

The data's semantic meaning is empirically established through encoding, which requires decisions about how to select and convert observations of elements of the real system into elements of the formal system (arrow 2 in Figure 5). To enable formal analyses, these conversion decisions must also consider syntactic relations that are identifiable in the selected indications of the real study system (arrow 1) and relevant to the research question (Uher, 2019). Qualitative differences (e.g., gender), rank-order differences (e.g., shoe sizes) or countable quantitative differences (e.g., finger rings) may be straightforwardly encoded into nominal, ordinal and ratio variables using isomorphic mapping relations as stipulated in representational theory. Mostly, however, psychologists encounter highly variable dynamic observables, such as in verbal and non-verbal behaviours (e.g., speech, gestures), that may be best described in sets of fuzzy observables in which syntactic structures cannot be straightforwardly identified.

Necessarily, the syntactic relations assigned to the formal model (arrow 3 in Figure 5) are also informed by the formal manipulations that they enable (e.g., statistical analysis). But because the model is just a surrogate, its syntactic relations must be aligned to those that are identifiable in the observables of the real study system (arrow 1). This is crucial because observations constitute the only direct empirical evidence that can be obtained about the real study system. Observational raw data form the basis for modelling, in the formal system, the (presumed) non-observable relations in the real study system (for which different syntactic relations may be conceived) as well as for testing the model's appropriateness through coordination and calibration. Importantly, which observable indications and which of their syntactic relations are (possibly) informative about the non-observable measurands depends not on the indications' ease of observability but on the theories about the objects of research, the measurands, instruments and their empirical interactions. Selecting indications by desirable syntactic structures, as done in psychometric ‘instrument' design and stipulated by representational theory, leads to methodomorphism, result-dependent data generation—and eventually to biased ‘measurement' results.

Hence, whether a model's syntax and the statistical analyses performed on it (arrow 3 in Figure 5) are appropriate for, and thus informative about, the *empirical syntactic relations* in the real study system (arrow 1) depends on the model's empirically established relations to that real system (arrows 2 and 4). Ignoring the model's *empirical semantic relations*, such as by neglecting encoding, coordination and calibration, can lead to logical errors and inappropriate data analysis. For example, students sometimes analyse means and standard deviations for data on persons' gender (encoded, e.g., as ‘1', ‘0' or ‘1', ‘2', ‘3') by taking them for ratio rather than nominal values. Thus, they ignore their empirical semantics of their data, established during data generation, and assign a different syntax to them. Syntactic mismatches between real and formal system also occur when researchers encode the verbal answer values of Likert ‘scales' (‘instrument' indications) in numerical scores and assign to them desired syntactic relations (e.g., order, interval). This ignores the empirical semantics that the researchers themselves establish by making these assignments. Specifically, what justifies the assumption that “agree” (encoded as ‘4') reflects more than “disagree” (encoded as ‘2')? How can we assume that “neither disagree, nor agree” (encoded as ‘3')—thus, having no opinion or finding the item not applicable—constitutes more than “strongly disagree” (encoded as ‘1')? Given the verbal answer categories' logico-semantic meanings, it is no wonder that raters interpret these not as reflecting order or interval relations but only as categorically—thus, qualitatively (nominally)—different (Uher, 2018a, 2022a, 2023a).

In sum, *data always have, at once, semantic and syntactic relations*. Their semantic relations are established through coordinated empirical relations to the study phenomena. These determine which syntactic relations can be assigned to the data to appropriately represent those identifiable in the real system, thus also enabling their calibration.

Establishing the data's empirical semantics is complexified by human language. Its peculiarities first enable the use of language-based methods in empirical research, but they also obscure psychology's measurement problem.

### 3.5 Natural language: Intuitive and ease of use obscures inherent complexities and common confusions

Language is an essential means for psychological research because psychical phenomena (e.g., thoughts and beliefs) are accessible only by the individual itself, and they can be accessed in others (e.g., research participants) only through language (Uher, 2016a; Valsiner, 2007; Vygotsky, 1962). In everyday life, we use language to exchange with others intuitively and without much reflection. Yet, this ease of use often leads us to overlook unparalleled complexities that challenge empirical research, especially measurement.

#### 3.5.1 Language and mind: Different yet inseparable systems

Language, as we have seen, is a complex sign system. It involves physical carriers (signifiers; e.g., spoken or written words) that stand for and refer to something else (referents), which establishes their semantic meanings. The rules underlying the semantics and syntax (e.g., grammar) of language are construals of human minds. This also applies to pragmatics, the rules specifying the language's function in the context of social interaction (e.g., the communicating persons' intentions and beliefs). These rules enable competent language users to express complex meanings with some flexibility and in context-dependent ways as well as to infer the specific meaning that others may want to express verbally in a given context. The language rules established in a community feed back to the individuals who develop and use them by mediating and shaping both their intra-individual and inter-individual processes (e.g., feeling, thinking, memorising, interacting and negotiating) as well as the social institutions aimed at regulating these processes (e.g., family and government). Therefore, language and psyche are inseparable from one another while still constituting different kinds of phenomena (Peirce, 1958; Uher, 2015b,a, 2016a, 2018a; Valsiner, 2000, 2014a; Vygotsky, 1962).

We use our maternal language effortlessly and without being fully aware of its *inbuilt semantics, syntax and pragmatics*. This is because these complex rules form an inherent part of our psychical systems after we internalised them as children during our language socialisation. Therefore, as native speakers, we often struggle to explicate the rules that we intuitively use, and we are often surprised what rules foreign learners of our language can state. That is, we are *competent without comprehension* (Arnulf, 2020; Dennett, 2012). This entails that we rarely become aware of the inherently representational nature of language, which is built into its semantics. Indeed, in our minds, we do not perceive our words just as tokens of the objects to which they refer but as these objects themselves. This illusion makes language so highly functional in everyday life. Yet, it becomes apparent again in our struggles of learning a foreign language when we have to acquire new words as arbitrary tokens to refer to the things of the ‘world'. But once we have internalised (at last parts of) a given language's inbuilt semantics, we cannot easily blank it out anymore to enable reflection and reflexivity about the ways in which it modulates and shapes our thinking. This is what makes naming a word's font colour more difficult when that word itself denotes another colour (Stroop effect)—unless we do not know the language, then the task is easy.

Therefore, we often forget that semantic relations are just in our minds, linking our words and thoughts seamlessly with the objects to which they refer. As Alan Watts stated:

“When I use the word *thinking*, I mean precisely that process of translating what is going on in nature into … symbols … [U]sing symbols and using conscious intelligence has proved very useful to us. It has given us such technology as we have; but at the same time, it has proved too much of a good thing. At the same time, we've become so fascinated with it that we confuse the world as it is with the world as it is thought about, talked about, and figured about—the world as it is described. The difference between these two is vast…” (italics as in original; Watts and Watts, 1996, p. 26).

Our ability to use the inbuilt semantics of our natural language intuitively and with ease, lets us often overlook its representational nature and confuse our words with ‘reality'.

#### 3.5.2 The map is not the territory, the model is not reality, the word is not the thing

Korzybski (1933) established *general semantics*—the study of language as a representation of ‘reality'. In his critique of traditional assumptions about language, he illustrated the distinction between a real object and its formal representation by stating that

“A map is not the territory it represents, but, if correct, it has a similar structure to the territory, which accounts for its usefulness” (Korzybski, 1933, p. 58).

Korzybski used the map–territory relation to illustrate the distinction between our perceptions or beliefs of something and the actual ‘reality' of it. Specifically, a map of a city is not that city in itself. Reading a map is not the same as walking the streets. Maps depict in abstract symbolic ways only those parts of a territory that are seen as relevant for some purpose (encoding, arrow 2 in Figure 1). Therefore, we can establish for the same territory different maps (e.g., road maps, geographical or political maps). That is, maps are reduced semiotic representations. All maps are limited. They may be incomplete or outdated. ‘Reality' may change (e.g., closed roads). Moreover, using maps requires interpretation (decoding, arrow 4), which may involve errors. Therefore, our maps of some ‘reality' (arrow 3) neither are that ‘reality' in itself (arrow 1) nor can they exactly match that ‘reality' (arrows 2 and 4).

Korzybski (1933) highlighted that we tend to mistake our conceptual models of ‘reality' for that ‘reality' in itself. This occurs when we ignore that the *word is not the thing*, the *abstraction of something is not that something in itself* —and thus, also, that the *theory is not what it describes* and that the *data are not the study phenomena* for which they stand (Uher, 2018a, 2021a, 2022b). As Alan Watts put it more vividly,

“symbols bear the same relation to the real world that money bears to wealth. You cannot quench anybody's thirst with the word ‘water' just as you cannot eat a dollar bill and derive nutrition.” “Money simply represents wealth in rather the same way that the menu represents the dinner^16^” (Watts and Watts, 1996).

Korzybski warned of the logical fallacies that ensue when the model is mistaken for ‘reality'. These occur not just in everyday life but also in science. In psychology, for example, latent variables that were statistically derived in a formal (e.g., factor analytical) model (arrow 3 in Figure 3) are often interpreted as ‘traits', ‘psycho-physical mechanisms' or ‘personality factors' that causally underlie individuals' behaviours, thoughts and feelings (arrow 1; Uher, 2013, 2018b, 2022b). In psychological jargon, the term ‘data' is often used to denote both the study phenomena (e.g., in individuals) and the formally encoded information about them (e.g., on spreadsheet; Uher, 2021a). The term ‘variables', as well, often denotes not just parts of formal models but also the modelled real objects themselves (Danziger and Dzinas, 1997; Maraun and Gabriel, 2013; Maraun and Halpin, 2008; Uher, 2021d,c). The confusion of the model with ‘reality' is also reflected in the notions that we would study ‘correlated behaviours' or ‘measure variables' as well as in the demand to grant “a serious ontological status to variables” (Borsboom, 2008, p. 41). Conflated jargon promotes such confusions because it leads researchers to neglect a formal system's empirically established semantics, which defines its relations to the real system—and thus, these systems' epistemic separation.

In sum, language and its conventional rules are construals of human minds, which, at the same time, mediate and shape individuals' psychical processes. Its intuitive and ease of use enables but also obscures its inherently representational function, leading to common confusions between words and the ‘reality' that they denote. When using language-based ‘instruments', these challenges are incorporated directly into psychological ‘measurement'.

### 3.6 Language-based ‘scales' obscure the measurement process

Psychological ‘measurement' is unthinkable without everyday language. It relies on the idea that any phenomenon of interest can be empirically studied, and even ‘measured', as long as it can be verbally described. Accordingly, rating ‘scales' comprise brief verbal descriptions of the phenomena with which *raters*—the persons using these ‘instruments'—are assumed to interact. While efficient and easy to use, modelling this process is intricate.

#### 3.6.1 Obscured distinctions between psychical phenomena, language-based ‘instruments' and formal models

In physical measurement, all elements of the real study system—the objects studied, those used as measuring instruments, their lawful empirical interactions and the indications thus-produced—are all of physical nature. The model that semiotically represents selected information about them, however, is formal, thus non-physical (Figure 2). In psychology, by contrast, real and formal system cannot be easily distinguished. Psychical phenomena (e.g., intellectual abilities, beliefs) are non-physical, abstract and represent information—just as the formal models developed about them. Language, here used as method and ‘instrument', is a complex sign system to communicate information—thus, a formal system as well. These peculiarities complicate the epistemically necessary distinction between the real and the formal study system. It also blurs, within the real system, the distinction between the phenomena studied and those used as ‘instruments' for studying them. This complicates the conceptualisation of how the ‘instruments' can be used in a given method to produce information about the study phenomena—*psychology's measurement problem*.

These epistemically necessary distinctions are further hindered by the ambiguous use of the term ‘scale' in psychology. On the one hand, it refers to Stevens' (1946) concept of ‘measurement scales' which defines variables with specific mathematical properties (e.g., ordinal, interval and ratio)—thus, structures of formal models. On the other hand, the term ‘scales' denotes the ‘instruments' that enable empirical interactions with the measurands, just like physical measuring devices (e.g., weighing scale; Uher, 2022a). Formal scale and physical scale, however, although coordinated and calibrated with one another, are epistemically distinct elements of measurement (Figure 2). In psychology, this distinction is obscured when the rating items serve both—as descriptions of the study phenomena in the verbal ‘scale' and as item variables in the formal model (Uher, 2018a). But raters interact only with the item statements of the ‘scales', not with the statistical models through which these were designed. So, what function do the item statements have when used as ‘instruments'?

#### 3.6.2 Specifying the phenomena to be ‘measured' through the inbuilt semantics of everyday language: Collective fields of meanings

Rating items categorise and describe the phenomena to be ‘measured'. Worded in everyday language, this enables lay persons to use rating ‘scales' with just minimal instruction and without any training. This differs fundamentally from many kinds of physical and behavioural measurement (Uher, 2018a, 2021c). Thus, psychologists capitalise on raters' and their own intuitive knowledge and use of natural language and its *inbuilt semantics*.

The inbuilt semantics of our words—their *conventional meanings*—are described in our dictionaries. Words can be grouped by their dictionary meanings and described in their semantic relations with other words using logic-based formalisms. These interrelations between words form *semantic networks*, which can be visualised in graphical networks. These networks describe common structures in the organisation of knowledge representations and information retrieval pathways that are socially shared by competent users of a given language (Arnulf et al., 2018; Pirnay-Dummer et al., 2012). In the semantic space of a language, a word's multi-dimensional associations with other words span a *field of meaning* (Rosenbaum and Valsiner, 2011; Uher, 2018a, 2022b, 2023a).

The general semantic meaning that language users *collectively* construe for a word is derived and abstracted from the specific meanings that individual users *locally* construe for it in the specific contexts of its use. A ‘house', for example, may mean a building serving as family quarters, refuge or shelter, but also a dynasty (House of Windsor), governmental institution (House of Commons), gathering place for specified activities (coffee house), or a business organisation (publishing house). That is, words may refer to concrete observables (e.g., buildings)—thus, they have a primary literal meaning (*denotation*). But many words also often imply *interpretations and explanations* of their referents (e.g., regarding their purpose) or they may be used as metaphors (e.g., ‘house' as ‘dynasty'; Lakoff and Johnsen, 2003). Thus, words may also have additional non-literal meanings (*connotations*). These meanings are more abstract and socio-culturally construed and often cannot be easily traced back anymore to their formerly concrete references and contexts (Deutscher, 2006).

This also applies to psychology's study phenomena. Most behaviours possess various observable features and can therefore be interpreted differently regarding possibly associated psychical phenomena (e.g., different intentions or feelings; Shweder, 1977; Smedslund, 2004; Toomela, 2008; Uher, 2015d). Describing the act of taking an object as ‘finding', ‘exploring', ‘securing', ‘catching', ‘seizing', ‘grabbing' or ‘stealing' implies different interpretations regarding the actor's (presumed) goals and intentions in the given context. That is, behaviours can be described in their momentary and localised physical properties (Uher, 2016b). But their explanations can go well beyond the here-and-now and can invoke various interpretive perspectives. These all follow logical principles (Kelly, 1955; Smedslund, 2004) yet without being logically determined by the behavioural act itself (Shweder, 1977).

Many words also imply *normative evaluations*. As members of the same community, individuals are substantially similar to one another. Evaluating normativity therefore requires abstracting from commonalities and focussing instead on minor variations (e.g., behavioural, facial) that are informative for differentiating between (groups of) individuals. Promoted by social appraisal (e.g., valued, sanctioned) and putative explanations (e.g., innate, intentional), socially relevant variations are often exaggerated. Then they appear in people's minds to be larger than they actually are, thereby acquiring *salience* (Uher, 2013). Those salient variations that are considered most important in a language community may eventually become encoded in words (lexical hypothesis; Allport and Odbert, 1936; Galton, 1884).

All this entails that everyday language is replete with inferential assumptions, implicit connotations, socio-cultural valences, interpretations and putative explanations (Shweder, 1977; Smedslund, 2004). This allows rating items to be worded such that they refer to a broader range of phenomena and contexts that raters could consider as well as to capture raters' interpretation, explanation and normative appraisal of them (Uher, 2015c, 2018a, 2023a). This shows again key differences between physical measurement and the pragmatic quantifications used in psychology. They arise from the fact that psychology's focus is on the individual (subjective) and socio-cultural (inter-subjective) interpretations, explanations and appraisals of observable (and inferred non-observable) phenomena—thus, on the meanings that these have for individuals and communities. This differs from physics and metrology, which aim to explore just the phenomena and their relations in themselves but not also our human experience and apprehension of them (Uher, 2020b, 2021b; Wundt, 1896).

This also highlights the crucial role of persons in the use of rating ‘scales'.

#### 3.6.3 Psychology's measurement problem is left to raters' intuitive decisions and local interpretations of standardised rating ‘scales'

Physical measurement requires objects used as measuring instrument that lawfully interact with the objects of research, thereby producing an indication from which information about the object's measurand can be derived. By contrast, language-based ‘instruments' themselves cannot interact with anything. Language involves not lawful relations but rules, which must be known and applied by persons. That is, *language-based methods require interpretation*, which is always context-specific, and thus variable. Moreover, psychology's objects of research are (primarily) human beings and specific phenomena and properties that are accessible only by persons (e.g., intensity of feelings, strength of beliefs) or that are studied from their individual perspective (e.g., perceived frequency, ascribed intentionality or normativity of others' behaviours). Therefore, it requires persons (e.g., research participants, patients) to interpret and use rating ‘instruments' and to identify relevant study phenomena. These persons must also interact with and judge these phenomena for specific purposes and from specific interpretive perspectives, and they must visibly indicate the outcomes thus-produced on the rating ‘scale' (e.g., by ticking a box). Hence, in psychology, the real study system involves *complex interactions* that are executed by persons. These persons therefore play a crucial epistemic role in the ‘measurement' process.

**Figure 6:**
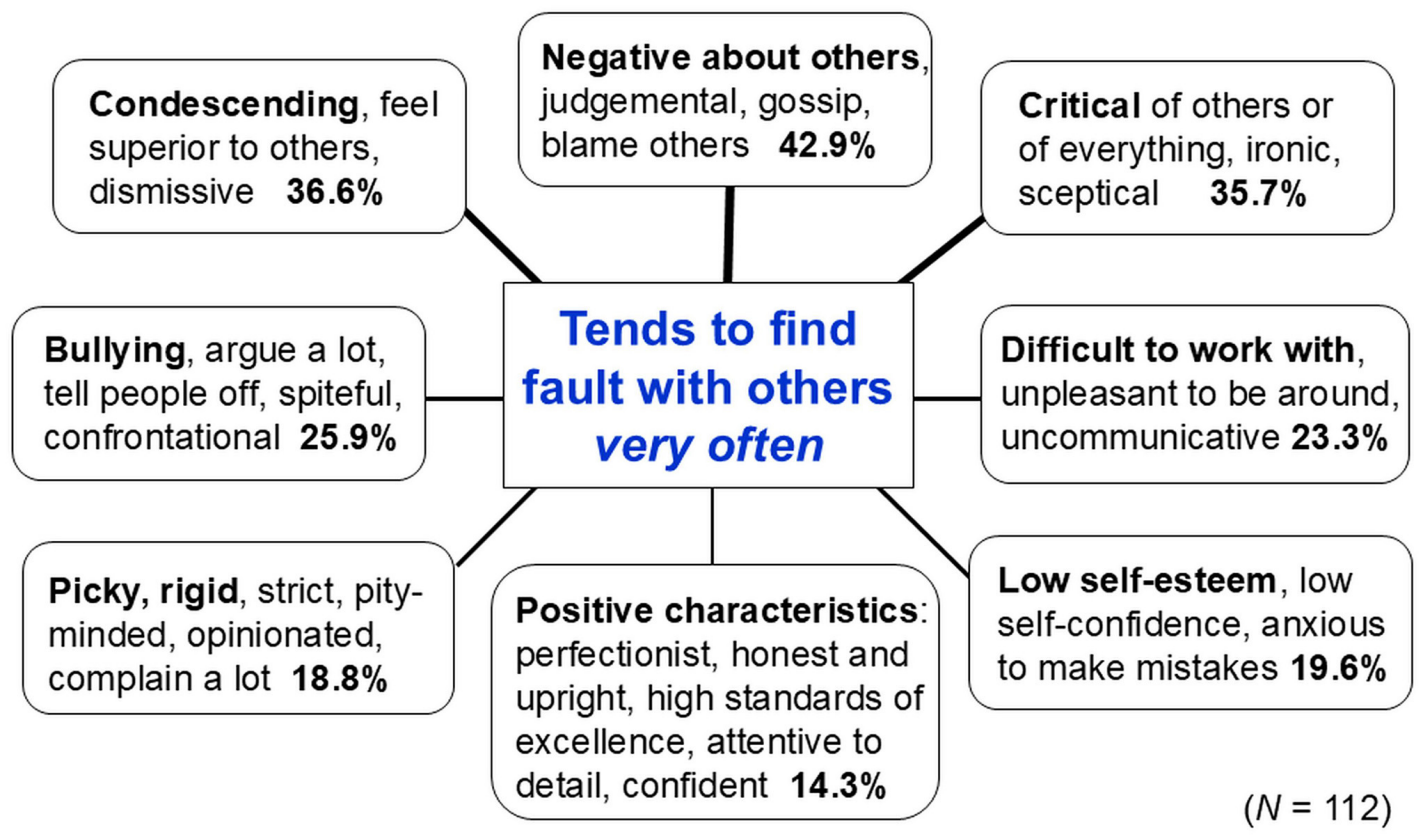
The inbuilt semantics of rating ‘scales': collective field of an item's local context-specific meanings. The collective general meaning of the item “tends to find fault with others”, used to operationalise the construct ‘Agreeableness' in the BFI-10. Its field of meaning is illustrated through the main themes that summarise the local context-specific item meanings that *N* = 112 participants construed for this item, described in their own words in terms of behaviours that a fictitious person scoring high on the item (indicated by ‘very often') would typically show. Percentages indicate the proportions of participants providing interpretations that are pertinent to a given theme (multiple nominations per person possible).

Language-based ‘scales' are standardised through identical wordings of items and answer categories and are therefore often thought to mean the same for all raters. This implies the assumption that all individuals interact with these ‘scales' in the same ways and produce indications that have the same meaning for everyone. But from the entire field of an item's general meaning, raters construe only a specific one that matches the context and specific interpretive perspective that they consider for a rating. Thus, they construe a *local meaning*. Figure 6 summarises the local meanings that 112 research participants independently construed for the item “tends to find fault with others” from a popular ‘personality' ‘scale'^17^. It depicts the broad field of meaning that this item statement collectively had for all raters but also the diversity of local meanings that they considered individually (Uher, 2018a, 2023a). That is, some raters read the item as “condescending,” others as “being picky, rigid,” still others as “having low self-esteem” or “being perfectionist, honest and upright” (Figure 6). On average, each rater considered only two different item meanings (*M* = 2.08; *SD* = 0.92; range = 1 to 5). No one considered the entire field of meaning. Thus, when used empirically by raters, standardised rating items have no unitary meanings.

Such variations in item interpretation, which occur both between and within individuals, were demonstrated also for other items of the same questionnaire (Uher, 2018a, 2023a) as well as in other studies (e.g., Arro, 2013; Lundmann and Villadsen, 2016; Rosenbaum and Valsiner, 2011; Uher and Visalberghi, 2016; Valsiner et al., 2005; Wagoner and Valsiner, 2005). The general dictionary meaning of rating items—their *inbuilt semantics*—can also be studied with artificial intelligence technologies.

#### 3.6.4 The inbuilt semantics of rating ‘scales': Natural language processing algorithms reveal its use by raters for mental short-cuts

*Natural language processing (NLP) algorithms* are types of artificial intelligence (AI) technologies to computationally analyse and process human language data. They are used either to identify specific structures and explicit rules in texts (‘understanding') or to produce texts from the algorithms identified (‘generative'). NLP algorithms dissect textual data sets (corpora) using statistical, mathematical or probabilistic methods (e.g., machine learning techniques). They analyse sentence structures (syntax) and keywords in order to identify or reproduce patterns and relations between words in sentences. NLP algorithms can be used, for example, to correct spelling (autocorrect), predict the next word given the preceding words (autocomplete), convert spoken words into written text (speech recognition), translate text from one language into another (machine translation) or extract the possible meaning (inbuilt semantics) of a sentence from its keywords and context or from the words' dictionary-based interpretation (content categorisation, automated text summarisation). To enable this, some NLP algorithms also rely on well-defined semantic and knowledge representations that are taken from linguistically established (previously hand-coded) dictionaries (Khurana et al., 2023). That is, NLP algorithms can formalise structures and explicit rules that underlie a given natural language and that can use these to analyse and generate texts.

Analyses of popular rating ‘scales' with NLP algorithms showed that the overlap in their items' inbuilt semantic meanings explained 60%−86% of the variance commonly found in ratings empirically obtained on these items (e.g., using factor analysis; Arnulf and Larsen, 2015; Arnulf et al., 2014). This sheds a new light on psychometrically established nomological networks. Traditionally, these are interpreted as sets of correlating item variables that encode the observable indicators (e.g., specific behaviours) through which a construct is operationalised (e.g., a ‘trait'). Instead, nomological networks may also largely reflect just the inbuilt semantic networks underlying the items' general (dictionary) meanings rather than any empirically derived structure in the phenomena described (Arnulf et al., 2024). Hence, ratings may reflect *likeness in semantic meaning* rather than *co-occurrence likelihood* of the phenomena described (Shweder and D'Andrade, 1980).

This was also demonstrated in multi-method studies. Associations of observer ratings on behaviour-descriptive items reflected their inbuilt semantic meanings but not the empirical patterns by which the described behaviours actually occurred in the same target individuals. Indeed, time-based measurements of functionally similar behaviours (e.g., different acts of aggression) showed only low to moderate internal consistency but substantial temporal consistency, thus indicating individual specificity (‘personality'). Observer ratings of the same target individuals on items describing the same behaviours, by contrast, were internally consistent—in line with their inbuilt semantic meanings (Uher et al., 2013a; Uher and Visalberghi, 2016). Thus, the inherently interpretive perspectives of rating items, reflecting socio-culturally ascribed valences and normativity, may influence and even bias perceptions and judgements of their observable referents (Shweder, 1977; Uher, 2022b; Vygotsky, 1962).

All this suggests that raters may use the inbuilt semantics of rating items also as mental short-cuts to simplify their rating task. Specifically, as thinking and learning agents, many raters do not fail to notice that rating ‘scales' commonly contain, in randomised order, items with similar content (a necessity for the psychometric analyses). Therefore, raters may focus on a few salient referents just for the first items on a ‘scale'. For any items perceived as ‘repetitive', however, they may generate their responses more efficiently by focussing just on their inbuilt semantic similarity instead of construing local meanings and considering specific referents for each single item anew (Uher, 2015c; Uher et al., 2013b).

Raters' locally construed meanings are commonly not inquired, however. Therefore, it remains unknown which specific phenomena and contexts they have considered in a given rating, from which specific perspectives (e.g., normative appraisal) they have judged them, and how they actually used the item ‘scales'. In consequence, the distinction between the objects studied and the objects used as ‘measuring instruments' *is left to intuitive decisions of raters*, who are commonly lay people. The intricate problem of conceptualising how the methods (and ‘instruments') interact with the study phenomena and can provide epistemically justifiable information about these phenomena—*psychology's measurement problem—*therefore remains undefined and unexplored (Uher, 2022b, 2023a). This ultimately obscures also the relations of the data and the formal model to the real phenomena under study.

#### 3.6.5 Researchers' focus on the inbuilt semantics of rating ‘scales' obscures the data's empirical semantics and syntax

**Figure 7:**
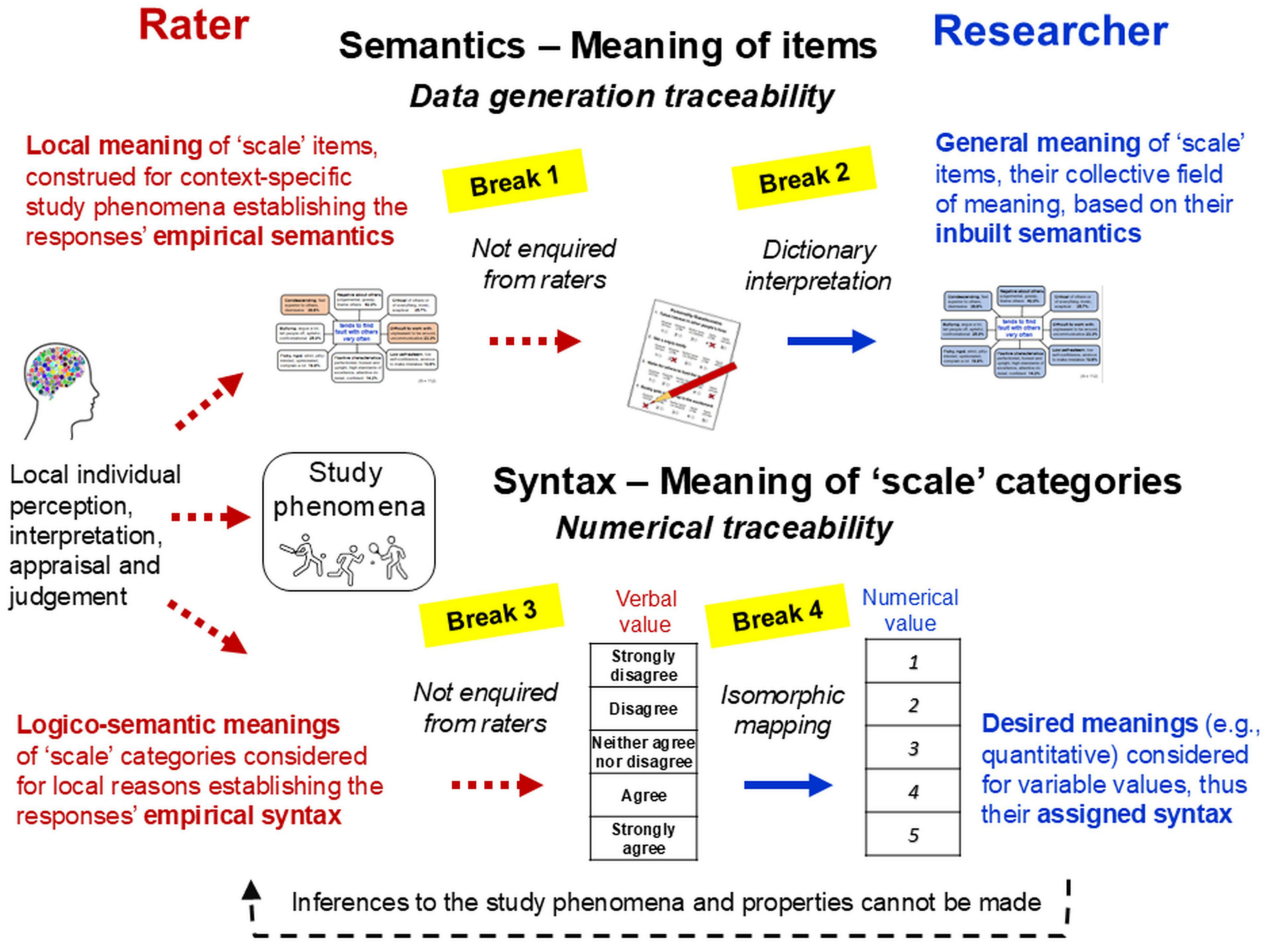
Four-fold break in data generation traceability and numerical traceability obscures the data's empirical semantics and syntax. Based on Uher (2018a, Figure 15).

Given that only the raters know how they have interpreted and used a ‘scale', only they can know what the rating data ultimately stand for and refer to—their *empirical semantics*. When encoding and analysing rating data, however, psychologists consider only the items' general meanings—their *inbuilt semantics*—ignoring the fact that raters consider for the *same* item *different* local meanings, *different* specific phenomena, *different* contexts and *different* interpretive perspectives. These *one–to–many relations in the data's empirical semantics* preclude tracing the data back to the real phenomena and contexts that raters have considered and judged and that their ticks on the ‘scales' were meant to indicate. But because raters' decisions are commonly not inquired—despite their crucial role in the data generation—these *breaks in data generation traceability* remain undetected (Figure 7).

Moreover, raters cannot indicate the outcomes of their interactions with the study phenomena (their judgements) in ways that they deem suitable for communicating them. Instead, raters can indicate their judgements only in a bounded set of verbal response categories that are specified a-priori by the researchers. We already discussed the syntactic mismatches that occur in agreement (Likert) ‘scales' between raters' primarily qualitative interpretation of ‘scale' categories (given their inbuilt logico-semantic meanings) and researchers' numerical encoding of them. Syntactic mismatches can also occur in frequency ‘scales' when raters are forced to use the *same* ‘scale' for *different* items—*regardless of the phenomena described*. Because different phenomena generally occur at different rates (e.g., chatting vs. shouting), this requires raters to indicate a broad range of quantities *flexibly* in the same ‘scale'. Raters can do so only by assigning *different* quantitative meanings to the *same* response value—a necessity that violates core ideas of measurement (Uher, 2022a). These *syntactic many–to–one relations* preclude that raters' indications on the ‘scale' can be traced back to the syntactic relations that they actually considered in the study phenomena. But these *breaks in the numerical traceability* of rating data remain undetected when raters' rationales for ticking ‘scale' boxes (indications) are not inquired and researchers consider instead only the syntactic relations that they themselves assign to the ‘scale' categories and their numerical encodings in the data (Figure 7).

In sum, using language-based ‘scales' to generate numerical data introduces several breaks in the semantic and syntactic relations between real and formal study system. But these breaks go unnoticed because quantitative psychologists do not consider raters' local interpretation and use of item ‘scales' but rely instead solely on the items' *inbuilt semantics* and on the syntax that they, as researchers, assign to raters' numerically encoded responses. Intuitive reliance on the inbuilt semantics of language-based methods also obscures the epistemically necessary distinction between the actual study phenomena and their verbal descriptions on the ‘instruments' and leaves it to raters' intuitive unknown decisions. In consequence, researchers cannot assess if their own decisions about how to encode raters' responses in numerical data (arrow 2; Figure 3) are appropriate (e.g., logical, consistent) for the real study phenomena. Researchers also cannot assess if their statistical analyses of the thus-generated data (arrow 3) as well as their interpretations of the results obtained are semantically and syntactically appropriate for the real study system (arrow 4) and can reveal epistemically justified information about its internal relations (arrow 1). That is, psychology's standard practice of generating quantitative data with rating ‘scales' fails to empirically establish the system of interrelated modelling relations that is required for measurement.

## 4 Statistics and language-based methods in quantitative psychology: Implications and future directions

Language is human's greatest invention (Deutscher, 2006). With words, we can refer to objects of consideration even in their absence (meaning), and although what we say or write (signifiers) typically bears no inherent relations (e.g., resemblance) to the objects referred (referents). This representational function of language—built into its semantics—is internalised in our minds and fundamental to our abstract thinking. However, we do not perceive our words just as tokens of the objects to which they refer but as these objects themselves. In our minds, we therefore easily mistake the word for the thing, the map for the territory, the menu for the food—the ‘world' as it is with the ‘world' as it is thought about and described. This also misguides our scientific thinking at times and leads to fundamental errors.

### 4.1 Psychologists' cardinal error: Failure to distinguish the ontic study phenomena from the epistemic means of their exploration

**Figure 8:**
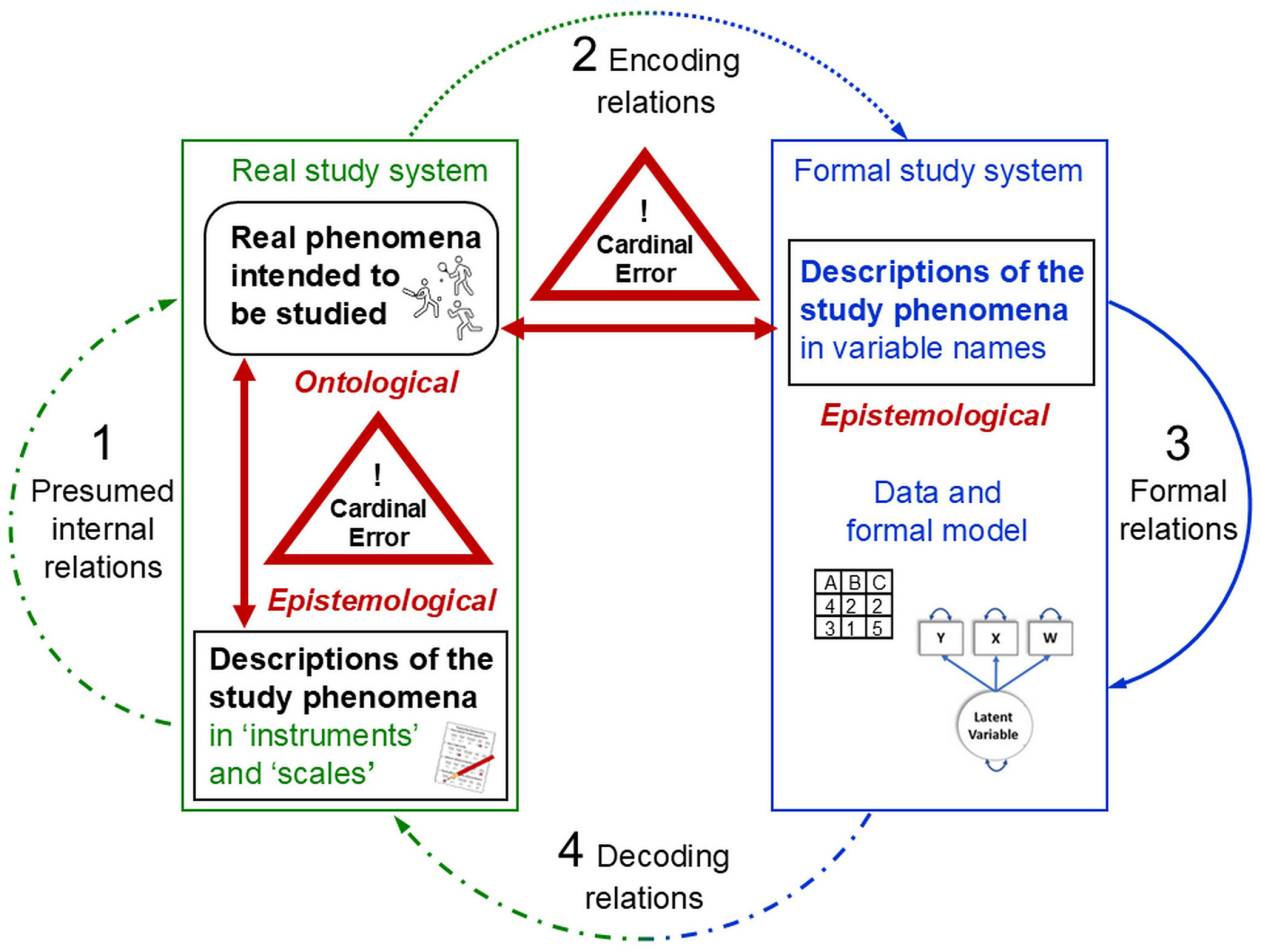
Psychologists' cardinal error: Failed distinction between ontological and epistemological concepts promoted by the inbuilt semantics of language-based methods.

Our tendency to mistake verbal descriptions for the phenomena described affects psychology in particular ways because we can access others' psychical phenomena never directly but only mediated through language. Unawareness of its inherently representational nature—its *inbuilt semantics*—often obscures the epistemic necessity to distinguish the study phenomena (e.g., raters' thoughts or feelings) from their verbal description in the language-based methods used for exploring these phenomena (e.g., item ‘scales', variable names). Failure to make this crucial distinction thus *confuses ontological with epistemological concepts*—therefore termed *psychologists' cardinal error* (Figure 8; Uher, 2022b, 2023a).

Psychologists' cardinal error can occur in various parts of the empirical research process. This logical error makes the distinction of disparate research elements and activities technically impossible and distorts basic concepts, methods and result interpretations (Uher, 2022b, 2023a)—such as in the processes required for measurement.

#### 4.1.1 The inbuilt semantics of language-based methods obscures the distinction between the ‘instruments' and the phenomena to be studied

The failure to conceptualise measurement processes in many psychological studies is often due to psychologists' cardinal error. This is because, when considering only their items' *inbuilt semantics*, psychologists often fail to distinguish the study phenomena's descriptions that are used as ‘instrument' from the described phenomena themselves that are intended to be studied (Figure 8). This error often underlies evaluations of face validity and content validity of psychometric ‘instruments'. It also underlies the widespread belief that any rating ‘scale' that is *nominally (by name)* associated with a study phenomenon could be a valid method for empirically studying it (e.g., ‘neuroticism scale'). This *nominalism* and *toolbox thinking* contribute to the proliferation of overlapping rating ‘scales' (e.g., various anxiety ‘scales') and of the likewise overlapping constructs that their items are meant to operationally define (Sechrest et al., 1996; Toomela and Valsiner, 2010; Uher, 2021b, 2022b).

#### 4.1.2 Mistaking judgements of verbal statements for measurements of the phenomena described: The risk of pseudo-empirical research

Psychologists' cardinal error also occurs when, through the *inbuilt* semantics of item ‘scales', researchers intuitively establish—in their minds—relations from their ‘instruments' to the study phenomena described. Their (and raters') mental construction of these relations (meanings) is necessary to specify the phenomena (referents) to be considered. But these mental relations only pre-structure their thinking—they *do not, themselves, implement any empirical relation to the real ‘world'*. Yet, because these relations are thought, they obscure the necessity to scrutinise what empirical connections are actually implemented in a study—and thus, what *empirical semantics* are established for the data thus-produced. ‘Personality' ratings, for example, enquire about habitual behaviours, which have necessarily already occurred in the past. Past events can be mentally (re-)construed. But traceable empirical interactions with those events, as required for measurement, can no longer be implemented.

In this way, the *inbuilt semantics* of language often leads researchers to misinterpret raters' judgements of verbal statements as measurements of the phenomena described in those statements (Figure 8). The necessity to conceptualise and empirically implement a coordinated and calibrated system of four interrelated modelling relations, as described in Rosen's general process scheme (Figures 1, 2), gets out of focus—and with it the actual phenomena under study. This entails the risk of replicating just verbal descriptions rather than exploring the real phenomena for which these are meant to stand (Baumeister et al., 2007; Cialdini, 2009; Doliński, 2018; Osborne-Crowley, 2020; Teigen, 2018; Uher, 2022b, 2023a; Wojciszke and Bocian, 2018). This puts quantitative psychology at risk of doing *pseudo-empirical* research, which mostly re-discovers what is necessarily true given the logico-semantic relations built into its language-based methods (Arnulf et al., 2024; Shweder, 1977; Shweder and D'Andrade, 1980; Smedslund et al., 2022; Smedslund, 1991, 2016b).

#### 4.1.3 Advancing just statistical methods and models: Creating a formal sphere disconnected from the ‘reality' to be explored

The focus on statistics leads quantitative psychologists to create formal spheres in which ever more sophisticated analyses and models can be developed. In the formal ‘world', there are no limits. This, however, ignores the epistemic necessity to empirically connect the formal models and data with the real study system, for which they serve only as surrogates—thus, to establish their *empirical semantics*. But the *inbuilt semantics* of the language terms that are used as data and variables in statistical models often lead psychologists to mistake the data for the phenomena and the models for the ‘reality' described—thus, to commit the cardinal error of confusing epistemological with ontological concepts. This confusion creates a data ‘world', a parallel universe of purely verbal representations but that has no traceable connections to the real ‘world'. Quantitative psychology then becomes a mere data science.

This empirical break leads many psychologists to overlook that *low replicability* is not just an issue of *epistemic uncertainty*, which could be remedied with more sophisticated procedures, but that it also reflects the study phenomena's *ontic indetermination*, variability, changeability and developmental nature. Psychology must advance concepts and empirical practices that are adapted to and appropriate for these peculiarities rather than focus only on what is possible in purely formal (e.g., statistical) systems. We cannot indulge in ever more complicated formal manipulations that have no counterparts in the ‘reality' that we aim to explore because this entails a proliferation of theories, constructs and supposed psychical phenomena for which there is little or no actual evidence. Ever more complicated statistics and their meticulous and transparent application (e.g., open science) therefore cannot tackle psychology's crises (e.g., in replicability, validity, generalisability), as currently believed, and but will only exacerbate them (Kellen et al., 2021; Uher, 2021b, 2022b; Uher et al., 2025).

#### 4.1.4 Statistics is not measurement: Psychology's pragmatic quantifications are numerical data with predictive power but without explanation

The common belief that statistics constitutes measurement is not just unwarranted. It is also misleading. In both everyday life and science, the term measurement implies that some part of ‘reality' is being quantified (e.g., some apples' weight). Measurement results are regarded as epistemically justified (e.g., we trust the shops' calibrated weighing scales; criterion 1) and publicly interpretable regarding their specific quantitative meaning for the object measured (e.g., ‘2kg' means the same weight everywhere; criterion 2). This differs from prices, customer ratings and other quantitative values that are attributed to some objects (e.g., apples) for some purposes and uses (e.g., trade, advertising). These pragmatic quantifications depend on considerations that go beyond the objects' specific properties and therefore vary across contexts and times, as does their specific quantitative meaning.

Quantitative psychologists' ‘measurement' jargon alludes to the *epistemic authority* of genuine measurement. This misleads the public (Barrett, 2003, 2018). It also leads researchers themselves to mistake their purely pragmatic research frameworks for the realist framework required for measurement, thereby misguiding concepts and theories.

Psychology's pragmatic quantifications (e.g., rating data, IQ scores) and statistical analyses (e.g., psychometrics) are useful for distinguishing individuals by their observable responses or performances as well as for making decisions and predictions on the basis of the differences and relations observed. But these approaches do not constitute measurement because they neither conceptualise nor empirically implement unbroken traceable connections between the results and the quantities to be measured (measurands) in the actual study phenomena. By adapting the results instead to statistically useful data structures (e.g., group differences), these approaches cannot explore the performances or responses observed for their underlying causes. These result-dependent methods thus preclude explorations of the *actual study phenomena*, such as what specific intellectual abilities individuals may use to solve a task or what they consider in their ratings.

In sum, psychology must address the *gap* that often exist between its numerical data and statistical models, on the one side, and its actual study phenomena and the specific quantities to be measured in them (measurands), on the other. To bridge this gap, it must advance *genuine analogues* of measurement.

### 4.2 Genuine analogues of measurement: Elaborating quantitative psychology's epistemological and methodological fundamentals

Rosen's process model conceptualises the system of interrelated modelling relations, which is generally necessary to develop formal models that are appropriate for exploring real study systems in empirical sciences (Figure 1). Psychology's challenge lies in the necessity to advance for this general process model specific concepts and practices that meet the peculiarities of its study phenomena and language-based methods. This is because quantitative analysis can be informative only when the system of modelling relations is also *empirically implemented*—both semantically and syntactically—rather than just presumed on the basis of the methods' *inbuilt semantics* and researchers' own syntactic assignments—that quantitative analysis can be informative at all.

#### 4.2.1 Metrological frameworks: Adaptations to psychological research are appropriate only on the more abstract philosophy-of-science level

Metrology enables accurate and precise measurement of quantities in non-living phenomena featuring invariant (unchangeable) relations. Interdisciplinary attempts to translate and apply metrological concepts rather directly to psychology (esp. psychometrics), however, overlook fundamental ontic differences in its complex study phenomena. These involve, amongst others, variable and context-dependent relations (e.g., many–to–one, many–to–many), novel emergent properties and dynamic multi-level feedback loops leading to continuous change and development of parts and wholes. Therefore, specific relations from observable phenomena (e.g., specific behaviours or test performances) to non-observable ones (e.g., specific intentions or intellectual abilities) that apply *to all individuals in all contexts and all times*—thus, that are invariant (one–to–one)—cannot be presumed. The study phenomena's *non-ergodicity* (non-equal synchronic and diachronic variations), as well, invalidates inferences from sample-level averages to measurands in single individuals.

Moreover, unlike metrology, psychology explores not just observable phenomena and their possibly underlying causes in themselves but also, and in particular, individuals' subjective and inter-subjective explanations, interpretations and appraisals of them. These multi-referential objects of research are conceptualised as *constructs* and require language-based methods for their exploration (Uher, 2022b, 2023a,b). Personality ratings, for example, were shown to be influenced by raters' knowledge of the phenomena and persons to be judged, raters' attitudes and relationships to them as well as raters' interpretation and use of the ‘scales' (e.g., items' inbuilt semantics, redundancy, social valences), leading to guessing, inattention and bias (e.g., centrality tendency, social desirability, stereotyping, halo effect; Kenny, 1994; Leising et al., 2025; Podsakoff et al., 2003; Shweder and D'Andrade, 1980; Tourangeau et al., 2000; Uher and Visalberghi, 2016; Uher et al., 2013b). That is, raters interact differently with the same ‘instrument', and even if they consider the same study phenomena in the same persons, they may invoke, in their ratings, different interpretational perspectives on them as well as indefinitely complex contexts. All this entails that rating data represent far more than just an observable ‘reality' and always reflect various strong influences *apart* from that concrete ‘reality' as well (Leising and Schilling, 2025).

That is, both psychology's complex study phenomena and its language-based ‘instruments' are *rich in interpretable information*. In metrological frameworks, by contrast, information is conceptualised only as the outcome of measurement, in the formal model, whereas the real study system comprises the physical objects studied, those used as instruments as well as their empirical interaction (Figure 2; Mari et al., 2021). Therefore, metrological concepts cannot account for different interpretive perspectives that persons (raters and researchers alike) can flexibly and intentionally take on the same object of research as well as on the same ‘instrument' and which are described with psychological constructs. Their conceptualisation is of no interest to metrology and physics but essential for psychology.

Still, as this article demonstrates, psychology can capitalise on metrology's theoretical fundamentals —just on far more abstract levels than interdisciplinary approaches can consider. This requires *transdisciplinary approaches*, as used here, to first make explicit and compare the different disciplines' basic ontological and epistemological presuppositions. This was a prerequisite for identifying the two abstract methodological principles (e.g., data generation traceability and numerical traceability) that implicitly underlie the metrological framework as well as for highlighting its direct conceptual connections to Rosen's general process scheme. The abstract philosophy of science perspective taken in transdisciplinarity is also essential to elaborate the ways in which concepts of physics and metrology, such as the problems of measurement and measurement coordination, can be meaningfully adapted to psychology to develop *genuine analogues of measurement* that are appropriate for its study phenomena's peculiarities (Uher, 2018a, 2019, 2020b, 2022a,b, 2023a, 2024).

#### 4.2.2 Epistemically justified evidence for psychological research and applied practice: Requirements and challenges

Researchers and practitioners in applied settings increasingly highlight that testing theories, hypotheses and the effectiveness of interventions as well as making decisions about individuals, such as in clinical, educational and legal settings, require epistemically justified evidence of the phenomena studied—which the result-dependent approaches of rating methods and psychometrics cannot provide (Barrett, 2003, 2018; Faust, 2012; Heine and Heene, 2024; Hobart et al., 2007; Mislevy, 2024; Rosenbaum and Valsiner, 2011; Truijens, 2017; Uher, 2022b, 2023a). It is therefore crucial to *remedy the empirical breaks* that often occur between psychology's study phenomena and its pertinent data and models (Figure 7). This requires elaborate concepts and approaches of *scientific representation* that allow for establishing unbroken traceable connections that are appropriate for mapping formal systems onto the peculiarities of psychology's study phenomena (arrow 2, Figure 2). To achieve this, psychology must also *advance its ontological and epistemological fundamentals* (Fahrenberg, 2013, 2015; Hartmann, 1964; Lundh, 2018; Poli, 2006; Uher, 2021b). It must also advance its *methodology*, such as to specify the abilities that data generation methods must have for capturing specific properties in the study phenomena and for establishing traceable relations with them (Uher, 2013, 2015c, 2018a; Valsiner, 2017).

All these fundamentals are underdeveloped in quantitative psychology. Much of its numerical data are still generated with a simple yet seriously flawed method, developed already a century ago but still lacking a conceptual foundation. The common belief that rating ‘scales' could enable standardised quantitative inquiries, implying that all individuals respond to standardised ‘verbal stimuli' in the same ways and produce ‘instrument' indications that allow for making straightforward inferences on the phenomena described, is unwarranted. It is surprising—if not paradoxical—that psychometricians claim that rating ‘scales' enable the ‘measurement' of individual variations while ignoring, at the same time, pronounced individual variations in the interpretation and use of these very same ‘scales'. Psychology's challenges arise from the peculiarities of its study phenomena (e.g., higher-order complexity, non-ergodicity) and language-based methods (e.g., inbuilt semantics). These make it impossible to establish coherent measurement models that enable inferences from standardised instrument indications to non-observable measurands that could be reliable and valid *for all individuals in all contexts and times*. That is, *psychology's problems of measurement, measurement coordination and calibration cannot be solved on the sample level*.

Meanwhile, psychology as a science in general is more advanced and acknowledges that researchers' own assumptions, beliefs, thinking and judgements can (unintentionally) influence their research methods, theories and findings (Danziger, 1997; Fahrenberg, 2013; Fleck, 1935; James, 1890; Marsico et al., 2015; Uher, 2013, 2015b; Weber, 1949). Quantitative psychology is still lacking behind these advancements (but see Jamieson et al., 2023). The common belief that quantitative methods could be generally more objective and free of subjectivity (‘scientific')—and thus, superior to others *per se* (*quantificationism*)—is erroneous (Strauch, 1976; Uher, 2022b). Quantitative psychology must acknowledge the fact that, given the peculiarities of its study phenomena and of the language-based methods required for their investigation, (lay) persons (e.g., participants, patients) play a crucial epistemic role in the data generation process. As an empirical science, psychology cannot build just on the researchers' own inferences from the inbuilt semantics of their language-based methods and on their own assignments of syntactic structures to their data and models. Such practices are prone to ethnocentric and egocentric biases on the researchers' part, leading to distorted theories and findings (Uher, 2015b, 2020a).

To justify the use of rating ‘scales' in psychological research and practice, it is of foremost importance to conceptualise and empirically explore how raters actually interpret and use these ‘instruments'. This is a prerequisite for establishing traceable, coordinated and calibrated connections from the study phenomena and known reference quantities to the generated results (data generation traceability, numerical traceability)—thus, for establishing genuine analogues of measurement (Figure 2; Uher, 2018a, 2019, 2022b, 2023a).

#### 4.2.3 Tackling psychology's problems of measurement coordination and calibration on the individual level: Empirical examples

Various lines of clinical research (e.g., on quality of life, chronic disease and therapeutic efficacy) already explored these problems under terms such as self-rated health (Fayers and Sprangers, 2002), patient-reported outcomes (PRO; Schwartz and Rapkin, 2004) and patient-centred measurement (PCM; Howard et al., 2024; McClimans, 2024; Pesudovs, 2006). They explicitly consider the fact that patients not only experience different symptoms, to different degrees and in different ways but also have diverse and changeable perspectives of their disease, treatment and quality of life. These researches consider that such complex study phenomena require for their description multi-referential conceptual systems (constructs) and language-based methods for their empirical investigation. Accordingly, they conceptualise in their methodological fundamentals the fact that patients' self-ratings involve perceptions, judgements, appraisals and also idiosyncratic criteria (Bosdet et al., 2021; Carr and Higginson, 2001; Kazdin, 2006; Schwartz and Rapkin, 2004; Truijens et al., 2019b).

This explicit conceptualisation is crucial to explain the frequent finding that changes in patients' self-ratings (e.g., pre-post treatment) often cannot be fully explained by actual changes in their health problems. Such *response shifts* pose challenges for evidence-based evaluations of clinical theories, treatments and therapies. They also question the utility of psychometric approaches for establishing the reliability and validity of assessment ‘scales'. Response shifts were shown to occur for various reasons. First, they arise from patients' context-specific local interpretation of rating ‘scales'. Furthermore, patients' interactions with the verbal descriptions of their symptoms on the ‘instruments' can change how they interpret their symptoms, how they understand and experience their own condition and thus, the meaning that these have for them. Response shifts may also be due to changes in patients' subjective frames of reference, the standards of comparison that they consider, the relative importance that they ascribe to symptoms, their recall and sampling of salient experiences, how they combine their appraisals when choosing an answer box on the ‘scale', and others (Desmet et al., 2021; Schwartz and Rapkin, 2004; Truijens et al., 2022; Vanier et al., 2021).

These findings illustrate why breaks in data generation traceability and numerical traceability occur when rating data are interpreted solely on the basis of their *inbuilt semantics* and *researcher-assigned syntax* (Figure 7). These and other lines of research demonstrated that raters' complex meaning-making processes must be considered to establish the *empirical semantics and syntax* of rating data—thus, their *epistemic validity*. Epistemic dialogue and other participative approaches involve both raters' first-person perspective and researchers' second-person perspective in order to probe into researchers' interpretation of raters' responses on standardised ‘scales'. This allows for establishing feedback loops between the real study system and its formal model (e.g., data) in order to coordinate and calibrate their *empirical semantic and syntactic relations* (Lahlou et al., 2015; McClimans, 2024; Truijens et al., 2019a; Uher, 2018a, 2022b, 2023a). These lines of research show that *psychology's problems of measurement and of measurement coordination can be tackled on the individual level*.

#### 4.2.4 Establishing the data's empirical semantics and syntax: Textual data from individuals' unrestricted verbal expressions vs. standardised rating data

To tackle these problems and to establish the data's epistemic validity, psychology must advance efficient methods for studying verbal descriptions that the studied *individuals themselves* find most appropriate to express their experiences. As George Kelly stated

“… each person seeks to communicate his [her] distress in the terms that make sense to him [her], but not necessarily in terms that make sense to others” (Kelly, 1969, p. 58).

**Figure 9:**
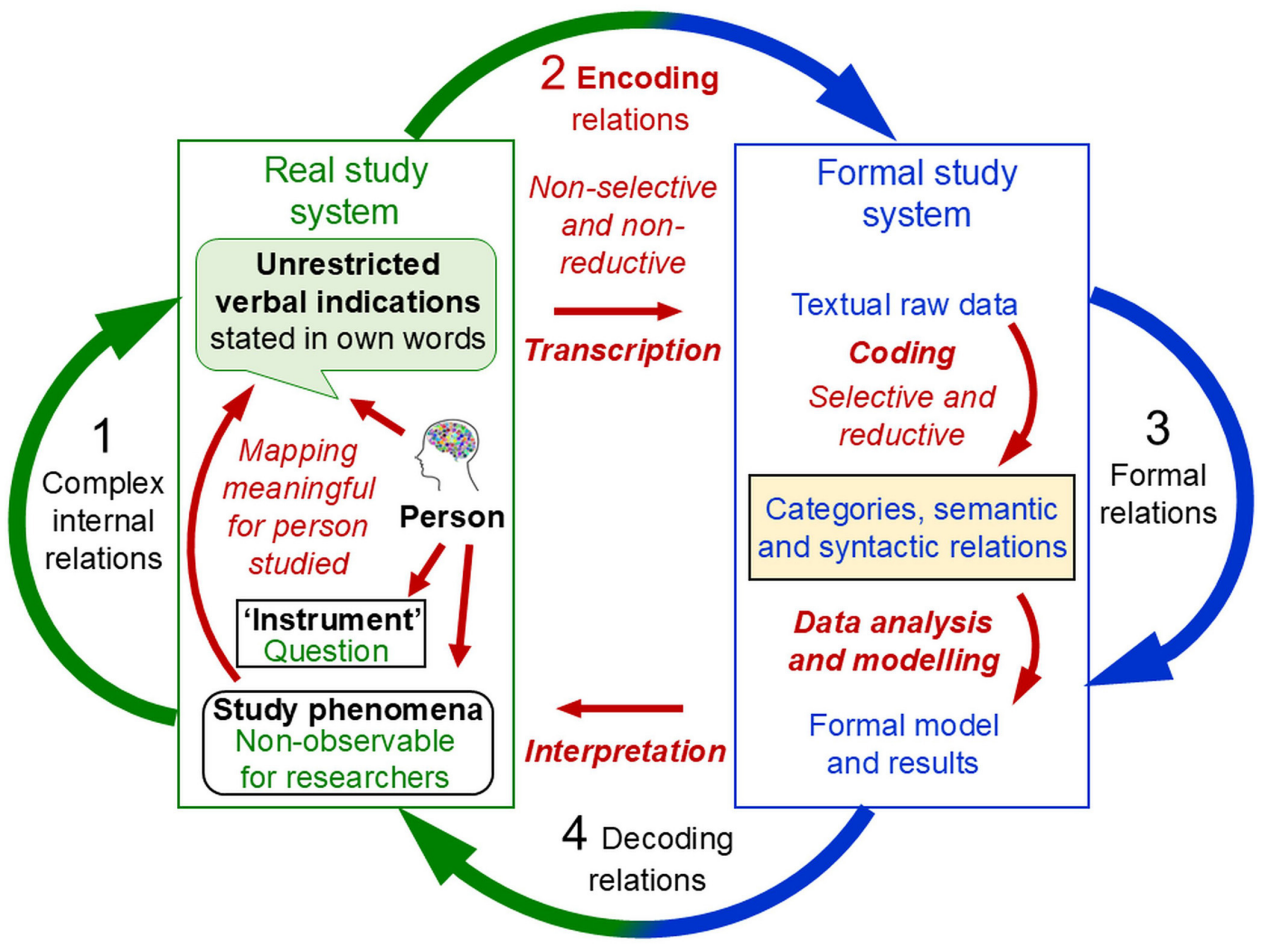
Establishing coherent systems of interrelated modelling relations through traceable encoding and analysis of individuals' unrestricted verbal expressions and ex post facto categorisation of study phenomena.

This requires methods for recording individuals' experiences and perspectives *without restricting their possibilities to verbally express themselves*. This insight is essential for conceptualising psychology's measurement problem. Specifically, individuals' interactions with a language-based ‘instrument' (e.g., survey question) and the phenomena under study (e.g., anxieties) as well as individuals' indications of the outcomes of these interactions must be *unrestricted* and *adaptable*. Such methods allow for establishing relations in the real study system that are *meaningful for these individuals themselves*. This is crucial for making the observable (verbal) indications that raters produce informative about the—for researchers—non-observable study phenomena and their occurrences to which only raters have access (arrow 1, Figure 9). This methodical requirement arises from the complex relations (e.g., many–to–one) in psychology's study phenomena. Metrological measurement models, by contrast, can deal only with unchangeable one–to–one relations of non-living nature, which can be identified through identically repeatable experiments. For this reason, the problems of coordination and calibration can be tackled on the sample level in metrology.

Individuals' indications, expressed in their own words, can be transcribed (e.g., verbatim) into textual data (or obtained from them in writing). This establishes documented, traceable and contextualised—yet *non-selective and non-reductive*—*encoding relations* between real and formal study system (arrow 2, Figure 9). The thus-generated textual raw data are then coded, whereby elements of individuals' encoded verbal statements are categorised into variables for further analysis. This establishes *selective and reductive coding relations*, which are likewise contextualised, unbroken, documented and traceable.^18^ Thus, crucially, the *selective reductive mapping of the real system's open domain to the closed sign system used as its model does not occur in the encoding relations between real and formal system* (arrow 2), as conceptualised in Rosen's system. Instead, it occurs in an *additional coding relation within the formal study system* (arrow 3). This additional step of formal analysis accounts for the study phenomena's complexity, which makes attempts for *a priori* or *ad hoc* selective reduction prone to reductionist biases on the researchers' part.

Methods of text analysis (e.g., data mining; content, thematic or discourse analysis^19^) provide strategies to systematically analyse textual data, such as for specific words, word sequences or word proximities but also for specific contents, recurrent themes, concepts or discursive elements, often coded in fuzzy categories. These can also be further analysed for their occurrences (e.g., frequencies, associations and configurations)—thus, for syntactic (e.g., quantitative) relations. *Transparency in the selection and reduction decisions* during coding and analysis makes the formal model and the results thus-derived as well as their quantitative meanings traceable to concrete occurrences of verbally described events. By implementing data generation traceability and numerical traceability through iterative coordination and calibration processes, the model's empirical semantics and syntax are established—thus, *genuine* analogues of measurement (Uher, 2022a,b, 2023a).

The known challenges of some of these text analyses (e.g., coding biases, limited generalisability) testify to the complexity of the analytical and interpretational decisions, which are always required to scientifically categorise—thus, to selectively reduce and semiotically represent—psychology's complex study phenomena and to identify meaningful syntactic relations in them. These challenges become directly apparent because, in these methods, they are dealt with in the *formal study system*, where they can be explored in *documented traceable* ways *by the researchers themselves* (arrow 3, Figure 9). This also means that information about the study phenomena, as verbally described by the individuals experiencing them (arrow 1) and textually encoded in the formal study system (arrow 2), is scientifically categorised *ex post facto*—after the events to be studied have occurred in the real system. This is essential because, in complex and context-dependent phenomena, it cannot be predicted which specific events may occur. For this reason, data generated with open-ended response formats or participatory procedures can provide rich and in-depth insights into human experience, as clinical research has demonstrated (e.g., on response shifts).

Conceptualising the measurement problem for rating methods, by contrast, reveals a very different process. For ratings, researchers categorise their study phenomena aligned to their research questions and own preconceived ideas *ex ante*—before knowing which specific events of interest may actually occur in the real system studied (e.g., individuals). Researchers verbally describe these categories in statements whose general meaning derives from their *inbuilt semantics*—because no specific events to be described have yet occurred. These *ex ante* categorisations, which also serve as standardised ‘instrument' indications (e.g., items), therefore need not be meaningful or even relevant to describe raters' concrete experiences and perspectives. Left without other options for expressing themselves, raters must adapt their interactions and judgements to the rating ‘scale' provided, thereby producing indications that are less informative, if at all, about the study phenomena and raters' views on them. This entails several breaks in traceability (Figure 7).

**Figure 10:**
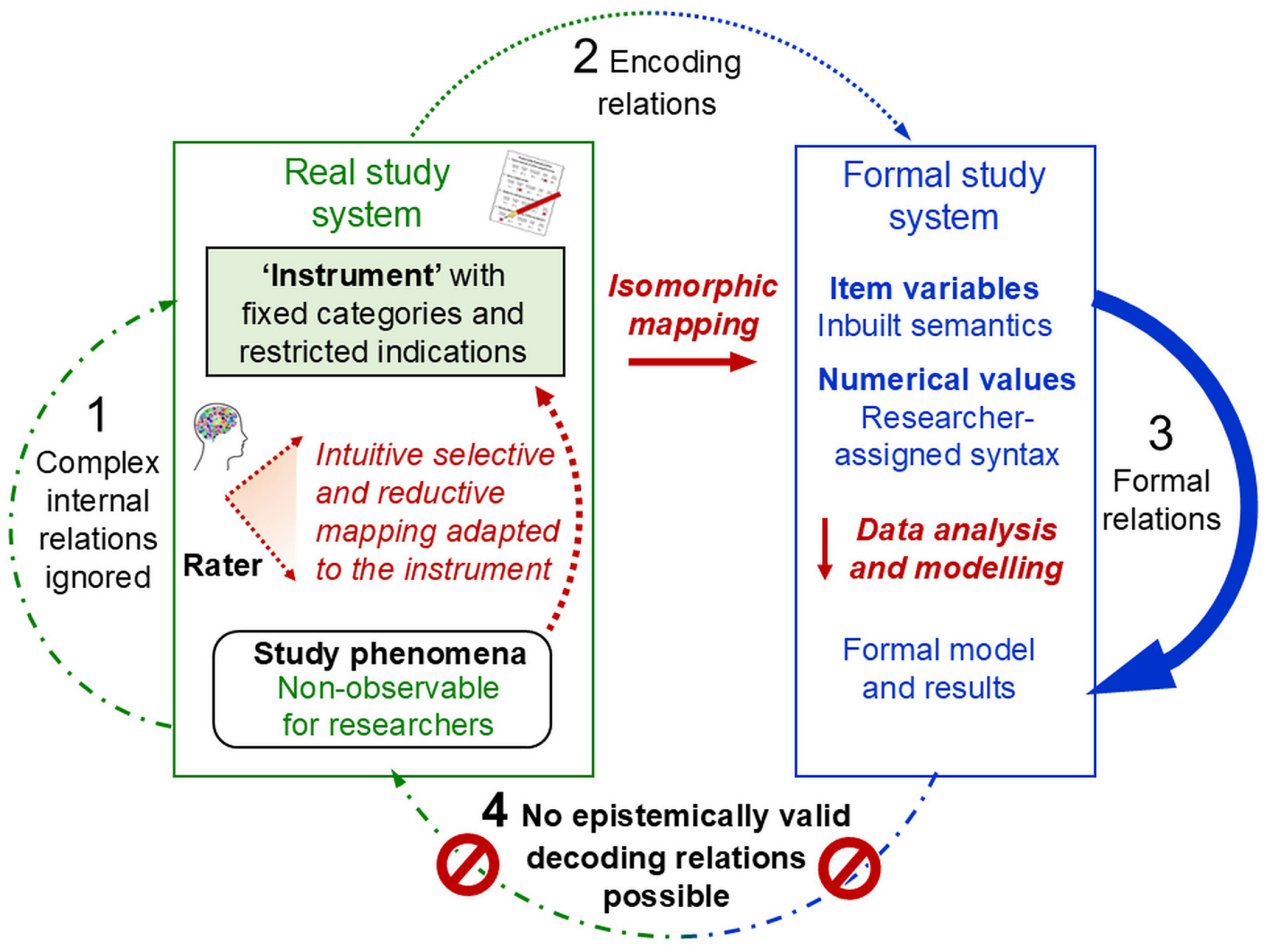
Rating methods: Ex ante categorisation of study phenomena and restricted instrument indications leave the crucial selective reduction decisions to raters' undocumented and non-traceable considerations.

Yet, these breaks do not become apparent because the intricate decisions of how to relate the study phenomena's structures and occurrences to the fixed ‘instrument' indications, both semantically and syntactically, are left to raters' intuitive decisions. Raters construe local meanings for standardised ‘scales' to make them meaningful for their specific experiences and contexts. But how specifically the single raters interact with the methods (‘instruments') and the study phenomena and thus, in what ways their observable indications can provide epistemically justifiable information about these phenomena remains *undocumented* and *non-traceable*. These relations are complex, variable, context-dependent and changeable. Therefore, they cannot be studied experimentally (unlike the one–to–one relations studied in metrology). Thus, in ratings, the *selective reductive mapping of the study phenomena's open domain to a closed sign system already occurs in the real study system*, inaccessible to researchers (arrow 1, Figure 10). This masks the tremendous challenges involved in the selective reduction of psychology's study phenomena. Moreover, this closed sign system itself (e.g., item statements) is aligned not to the specific events to be studied, as these have not yet occurred (*ex ante*), but to researchers' own preconceived ideas and study questions.

Researchers then encode raters' chosen indications using isomorphic mapping relations into rating data (arrow 2, Figure 10). Each standardised item statement is mapped to one item variable and interpreted regarding the general meaning of its *inbuilt semantics*. Raters' chosen answer boxes are rigidly encoded into predefined numerical values to which researchers attribute a desired syntax (e.g., quantitative meaning). As we have seen, this operationalist procedure introduces further breaks in the *empirical semantic and syntactic relations* between the rating data and the actual study phenomena (Figure 7). But these breaks often go unnoticed because, for rating methods, reporting standards demand traceability (transparency) only for the research design and statistical analyses (Appelbaum et al., 2018). But they do not also demand the data variables and values to be traceable back to occurrences of the study phenomena (as required, e.g., for ethological observations or software-based coding of behaviour). Therefore, rating methods preclude the conceptualisation and empirical implementation of coherent systems of interrelated modelling relations—and thus, of genuine analogues of measurement (Uher, 2018a, 2022b, 2023a).

In sum, psychology must invest more efforts to establish the epistemic validity of its data and models. These efforts can benefit from the advances made in artificial intelligence.

### 4.3 Artificial intelligence: Language algorithms can support psychological research but also perpetuate psychologists' cardinal error

Psychology's language-based research can capitalise on the powerful artificial intelligence (AI) technologies that are modelling human language and that are now available at large scale—especially NLP algorithms (Section 3.6.4) and *large language models (LLMs)*. These deep learning machines capitalise on the foundations of NLPs but build their own internal implicit algorithms from processing vast textual data sets (e.g., books and websites). This extensive training enables LLMs to identify, predict and generate patterns and relations in human languages with higher adaptability, coherence and contextual relevance than previous NLPs. Therefore, they can ‘understand' complex context, generate human-quality texts with human-like fluency and ‘converse' in human-like fashion (e.g., ChatGPT).

These performances can meaningfully support psychological research. But they also trigger our deep-rooted natural tendency to attribute human characteristics to non-human entities (Hume, 1757). We focus on what appears to be human-like—that is, *anthropo-morphic*—but tend to ignore what is human-unlike (anthropo-centric biases type I and II (Uher, 2015b, 2020a). This anthropo-centrism profoundly shapes also how we perceive and engage with AI machines, thereby misleading our understanding of their capabilities and limitations (Yildiz, 2025). This applies in particular to the challenges and pitfalls inherent to language-based AI machines—especially those arising from their inbuilt semantics.

#### 4.3.1 Efficient transcription and analysis of individuals' local context-specific meanings expressed in their own words through NLP algorithms and LLMs

Language algorithms can be used to efficiently analyse individuals' unrestricted verbal expressions—from transcription to the extraction of semantic and syntactic relations in documented traceable ways. Clinical researchers again pioneered in advancing methods for capturing and analysing the complexity of individuals' health conditions. They showed how patients' responses to well-prompted open-ended questions, expressed in their own words, can be analysed using machine learning techniques of NLP algorithms and LLMs. Their enhanced capabilities for analysing language context enabled more detailed and more accurate assessments of patients' heterogeneous and complex mental health conditions than psychometric ‘scales'—while also being individualised and efficient. Algorithm-based categorisations of open-ended self-descriptions discriminated even better between persons diagnosed with specific clinical conditions (e.g., anxiety, depression) and healthy persons than did pertinent self-ratings—although psychometric ‘scales' are statistically designed and selected for enabling such discriminations reliably (Islam and Layek, 2023; Kerz et al., 2023; Kjell K. et al., 2021; Kjell O. et al., 2021; Sikström et al., 2023; Tabesh et al., 2025).

Hence, NLP algorithms and LLMs can be used to efficiently analyse individuals' local context-specific meanings, expressed in their own words, and to extract, summarise and categorise their general meanings using the AI models' *inbuilt semantics*. Their algorithmic parameters can also extract *syntactical* (e.g., quantitative) information (e.g., frequencies, associations) to enable further analysis of the identified categories (e.g., group comparisons). This procedure implements documented and traceable modelling relations between individuals' verbally described experiences (real study system) and the coded data and models about them (formal study system). This allows for establishing the results' *empirical semantics and syntax*—thus, their epistemic validity as required for genuine analogues of measurement. Proponents of rating methods, by contrast, still adhere to the inverse—yet epistemically invalid—procedure and therefore use language algorithms for other purposes.

#### 4.3.2 Designing rating ‘scales' with language algorithms cannot establish the data's empirical semantics and syntax as needed for genuine measurement analogues

Quantitative psychologists increasingly use NLPs and LLMs to design or improve psychometric ‘scales'. Some aim to reduce the semantic overlap between ‘scales' (Huang et al., 2025), to improve the content validity for specific constructs (Hernandez and Nie, 2023) or to tackle the incommensurability of constructs and operationalisations across studies (Wulff and Mata, 2023). Others aim to improve the prediction of human interpretation for more “robust, objective assessments” and to “enhance the scientific rigour” of psychometric tests (Milano et al., 2025). Thus, the *inbuilt semantics* of language algorithms is used to predefine categorisations of study phenomena (standardised statements). Their general meanings then serve as both ‘instruments' and item variables to explore individuals' local context-specific meanings of their experiences and views on them. But as we have seen, these result-dependent procedures lead to several breaks in the thus-generated data's and models' traceability back to the phenomena studied in the real system (Figure 7). That is, they fail to establish the resulting model's *empirical semantics and syntax*—its *epistemic validity*.

This increasingly popular approach corresponds to creating a city map using well-established cartographic symbols and structures (e.g., for roads, buildings) yet *without mapping it empirically* onto a real city. It creates not a *map* but just an image of a city that may but need not exist as depicted. This is also like polishing the food descriptions on a restaurant's menu on the basis of what can generally be cooked, regardless of what dishes are actually cooked on a given day. Using AI algorithms of human languages to design psychometric ‘instruments' cannot remedy the empirical breaks between real and formal study system.

Moreover, the basic idea is not new. Lexical approaches in differential psychology capitalise on the inbuilt semantics of natural languages, building on the assumption that those individual differences that are most salient will eventually become encoded in words. This *lexical hypothesis* (Galton, 1884; Klages, 1926) provided a stringent rationale for using the person-descriptive words in our natural languages to identify a few major dimensions of individual differences that are considered most important in folk psychology. This rationale underlies many popular ‘personality' models developed over the last century (e.g., Big Five, 16PF; HEXACO; Allport and Odbert, 1936; John et al., 1988; Uher, 2013, 2015c, 2018b).

Despite its enormous importance for taxonomic research, however, the lexical hypothesis itself remained untested—even 141 years after its first articulation (Toomela, 2010; Uher, 2013; Westen, 1996). Still little is known about what specifically gets encoded in a language and how, what may be missed out and why. Humans invented an estimated 31,000 languages, of which only some 7,000 still exist (Crystal, 2000). Their vocabularies differ in what they allow us to describe. Their rules are extremely diverse, involving not just different scripts and speech patterns (signifiers) but also different rules that enable and enforce the communication of different types of information. In different languages, for example, communicators *either cannot or must indicate*—such as by modifying word endings—the reference to time (tense) and/or the extension over time (aspect); the agent (voice), state of completion and/or intentionality of actions; the grammatical gender and/or number of persons, objects, their attributes and/or actions; the syntactic function of persons, objects and events in a sentence (declension); the communicator's relation to the recipient, intention for communicating and/or source of the information communicated (e.g., whether from own observation, hearsay and/or inference), and others (Boroditsky, 2018; Deutscher, 2006, 2010).

That is, everyday language encodes everyday knowledge with all its socio-cultural biases and insufficiencies. If the everyday knowledges encoded in the semantics, syntaxes and pragmatics of our natural languages were epistemically valid and sufficiently accurate to describe and explain the structures and functions of human psyche, behaviour and society, then language scientists (e.g., linguists, philologists) would have long accomplished this task. But given the tremendous differences between languages, this strategy is epistemically not justified. Indeed, most AI technologies were developed in English. English is a mongrel language whose grammar was simplified already during the Mediaeval ages, when it was synthesised from Old English, Welsh, Gaelic, Danish, Norse, French, Old German and other languages. A focus on English-language algorithms will inevitably introduce ethno-centric biases, as happened before when Anglo-American ‘personality' models (e.g., Five Factor Model) were claimed to be ‘universally' valid for all human cultures (Uher, 2015c, 2018b).

Language algorithms are trained to identify and *re-*produce structures in human language—that is, they are modelling *human-produced* text or speech. But they cannot and do not establish relations (meanings) from the written or spoken sentences (signifiers) to the real ‘world' (referents) that is being described in the language they are modelling. It is us, as humans, who construe, in our minds, these semantic relations to the real ‘world' described (meanings). Meanings decay with individuals' minds (e.g., in dementia) and with their lives (Uher, 2015a). Therefore, languages die out with the persons using them (Crystal, 2000).

Language-based algorithms merely *re-*produce signifiers (words) and structures between them in ways that correspond to those that we use our languages. These structures were created through the efforts of past generations to mentally and semiotically represent the real ‘world' around us and to communicate about it. AI systems meanwhile mimic these human-built structures in such sophisticated ways that we can easily integrate them into our thinking. This makes us inclined to attribute to the machine our own thinking of the semantic relations, which are built into our language and internalised in our minds. But we tend to overlook the fact that it is us who are thinking these relations, not the machine. This becomes obvious when we look at texts generated in a language foreign to us. Without having internalised its semantics, we cannot make sense of what is written—not mentally relate it to what it stands for in the real ‘world' described. The machine cannot do this for us.

Our human abilities to immediately and effortlessly relate our language to the real ‘world' described often leads us to overlook the crucial difference between the study phenomena and the means of their exploration (e.g., descriptions). To avoid confusing ontological and epistemological concepts—psychologists' cardinal error—psychologists should have at least some basic knowledge of human language. This is also necessary to use language-based algorithms in epistemically justified ways to advance psychological research.

One of psychology's key challenges lies in the fact that it must necessarily rely on lay people's abilities and their everyday language. This requires engaging with the individuals studied rather than distancing ourselves ever more from them by studying just standardised abstract descriptions of collective meanings that are predefined by researchers or AI machines. A science of psychology should advance approaches and methods that are epistemically justified for exploring its study phenomena in the specifics and contexts of their occurrences. Therefore, we need to know how individuals use their natural language and relate it to the real ‘world' as they experience and see it in their given contexts. This knowledge will be crucial to systematically connect psychology's language-based data and formal models with the real-world phenomena that these are meant to represent and for which they serve only as surrogates—thus, to establish *genuine* analogues of measurement.

## Data Availability

The original contributions presented in the study are included in the article/supplementary material, further inquiries can be directed to the corresponding author.

## Footnotes

^1^Here, the terms psych*ical* and psych*ological* (from Greek *-logia* for body of knowledge) are distinguished to express the crucial distinction between ontological concepts describing the objects of research themselves (e.g., mental, emotional) and epistemological concepts describing the means for exploring these objects of research (see Section 4.1). This distinction is made in many languages (e.g., French, Italian, Spanish, German, Dutch, Greek, Russian, Norwegian, Swedish, Danish) but not commonly in the English (Lewin, 1936; Uher, 2021b, 2022b, 2023a).

^2^The terms ‘scales' and ‘instruments' are put in inverted commas because they do not enable all methodological functions that genuine measuring scales and instruments fulfil, as shown in this article and in Uher (2022a).

^3^*Interdisciplinarity* is the synergistic collaboration of several disciplines who work on a specific research objective or problem whose solution is beyond a single discipline's scope. It is aimed at synthesising and integrating perspectives, knowledge, theories and concepts, whereby approaches and methods are transferred between disciplines and integrated through the research topic into their disciplinary work. Often, interdisciplinary projects benefit just one of the disciplines involved but not the others, or at least not immediately (Russell, 2022; Uher, 2024).

^4^Coss-disciplinary, multi-disciplinary or inter-disciplinary collaborations are sometimes erroneously referred to as ‘transdisciplinary', ignoring the fundamental differences between them (Bernstein, 2015; Uher, 2024).

^5^There are two schools of transdisciplinarity. The present analyses build on *theoretical transdisciplinarity*. *Applied (practical) transdisciplinarity*, by contrast, is aimed less at developing theoretical frameworks and new forms of knowledge but more at understanding complex real-world problems and developing tangible solutions. It involves scholars from different disciplines but also political, social and economic actors as well as ordinary citizens with the aim of producing socially robust knowledge rather than merely reliable scientific knowledge (Uher, 2024).

^6^The TPS Paradigm is aimed at making explicit the basic presuppositions that different disciplines (e.g., biology, medicine, psychology, social sciences, physical sciences) make about research on individuals and their multi-layered ‘realities' considering phenomena from all domains of human life. These involve abiotic phenomena (e.g., non-living environment), biotic phenomena (e.g., physiology, behaviours), psychical phenomena (e.g., emotions, thoughts) and socio-cultural phenomena (e.g., culture, language), which are all merged in the single individual and its functioning and development but involve different layers of ‘reality'. This poses challenges for empirical inquiry because different kinds of phenomena require different ontologies, epistemologies, theories, methodologies and methods, which are based on different, even contradictory basic presuppositions (Uher, 2024). To provide conceptual fundamentals that are appropriate to tackle these challenges and to discover hidden connections between scientific disciplines and their knowledges, relevant established concepts from various sciences have been systematically integrated on the basis of their basic presuppositions and underlying rationales and complemented by novel ones. This enabled the development of three unitary frameworks that coherently build upon each other (therefore, it is termed a ‘paradigm'), that transcend disciplinary boundaries (therefore termed ‘transdisciplinary'), and that are aimed at making explicit the most basic assumptions made in a field (therefore termed philosophy-of-science). The *philosophical framework* comprises presuppositions for research on individuals (e.g., complexity, complementarity, anthropogenicity). The *metatheoretical framework* comprises, amongst others, metatheoretical definitions of various kinds of phenomena studied in individuals, differentiated by their modes of accessibility to humans (e.g., physiology, psyche, behaviour, sign systems like language; Uher, 2013, 2015b,c, 2016a,b, 2023b). It informs the *methodological framework*, which comprises, amongst others, classifications of data generation methods based on the modes of accessibility that they enable; basic principles, concepts and theories of measurement and quantification across the sciences demonstrated in empirical multi-method comparisons as well as critical analyses of the foundations of psychometrics and quantitative psychology: http://researchonindividuals.org.

^7^This is one of the most intricate and also variously defined problems of quantum physics. It arose because its micro-physical objects of research (e.g., electrons) cannot be made accessible to observers other than through their interactions with macro-physical instruments (e.g., detection screen). This entails challenges called the measurement problem. Simply put, quantum physicists sought to explain how the macro-physical instruments can provide information about micro-physical objects, thus what constitutes a measurement. This required the conceptual distinction between the object of research and its environment (incl. the instruments and methods of observation)—the *Heisenberg cut*. It also required explanation of the processes by which the micro-physical objects under study interact with the macro-physical objects used as measuring instruments, which however are debated still today (Atmanspacher, 1997; Hance and Hossenfelder, 2022; Heisenberg, 1927; von Neumann, 1955).

^8^Rosen himself (Rosen, 1985, 1999) and others refer to this process model solely as *modelling relation*. To highlight that it involves the coherent modelling of four interrelated modelling relations (arrows 1 to 4) and to pinpoint key distinctions to the statistical modelling of data, which concerns solely arrow 3 in Rosen's general model, I refer to his process model as a *system of interrelated modelling relations*.

^9^Also called *rules of correspondence* (Kaplan, 1964/2017; Margenau, 1950; Torgerson, 1958).

^10^Therefore, mere nominal categorisation should not be confused with measurement.

^11^Numerical traceability is the transdisciplinary term to denote—on more abstract levels of consideration—the basic principle underlying the concept of metrological traceability used in physical measurement (JCGM200:2012, 2012) in order to adapt it to the peculiarities of non-physical research.

^12^The standard unit of one kilogram, originally specified through artefacts, was recently defined in terms of natural constants using the Planck constant, speed of light and the Caesium atom's resonant frequency (BIPM, 2019).

^13^Invariance here refers to *what kinds of objects* are always related to one another (one–to–one rather than e.g., one–to–many), not how. Their specific relations may have quantitatively different forms (e.g., linear, non-linear).

^14^The theorems were first derived in ergodic theory, a branch of mathematics originating in statistical physics.

^15^*Transcription* is the process whereby DNA is copied into RNA, following lawful (inevitable, necessary) pairings between bases (e.g., cytosine with guanine) determined by their molecular structure. *Translation*, by contrast, is the process whereby specific RNA codons instruct the synthesis of specific proteins following rules, which are not inevitable and not necessary given the bases' molecular structure but arbitrary—they could also be otherwise.

^16^The menu–food metaphor was also used by Arnulf et al. (2024).

^17^Big Five 10-item short version (BFI-10; Rammstedt and John, 2007).

^18^These are specified, for example, in the methodological and epistemological foundations of a given method as well as in internationally agreed reporting standards (e.g., for so-called ‘qualitative' methods in Levitt et al., 2018).

^19^Some of these methods are commonly called ‘qualitative', as opposed to ‘quantitative' ones. This polarisation overlooks, however, that any quantity is always *of something*—a specific quality (Kaplan, 1964). Quantities are divisible properties of entities of the same kind—the same quality. Anything that is to be quantified must first be qualified in terms of the kind of entity that it is (Hartmann, 1964). Moreover, many so-called ‘qualitative' methods establish data generation traceability and numerical traceability, thus meet the epistemic criteria of measurement, whereas rating methods, commonly regarded as ‘quantitative', do not (Uher, 2022a,b, 2023a).
